# Toward a Principled Sampling Theory for Quasi-Orders

**DOI:** 10.3389/fpsyg.2016.01656

**Published:** 2016-11-29

**Authors:** Ali Ünlü, Martin Schrepp

**Affiliations:** ^1^Centre for International Student Assessment, Technical University of MunichMunich, Germany; ^2^SAP AGWalldorf, Germany

**Keywords:** discrete doubly inductive quasi-order construction, simple random sampling, stratified sampling, absolute rejection, resampling, item tree analysis, knowledge or learning space theory, representative random quasi-order

## Abstract

Quasi-orders, that is, reflexive and transitive binary relations, have numerous applications. In educational theories, the dependencies of mastery among the problems of a test can be modeled by quasi-orders. Methods such as item tree or Boolean analysis that mine for quasi-orders in empirical data are sensitive to the underlying quasi-order structure. These data mining techniques have to be compared based on extensive simulation studies, with unbiased samples of randomly generated quasi-orders at their basis. In this paper, we develop techniques that can provide the required quasi-order samples. We introduce a discrete doubly inductive procedure for incrementally constructing the set of all quasi-orders on a finite item set. A randomization of this deterministic procedure allows us to generate representative samples of random quasi-orders. With an outer level inductive algorithm, we consider the uniform random extensions of the trace quasi-orders to higher dimension. This is combined with an inner level inductive algorithm to correct the extensions that violate the transitivity property. The inner level correction step entails sampling biases. We propose three algorithms for bias correction and investigate them in simulation. It is evident that, on even up to 50 items, the new algorithms create close to representative quasi-order samples within acceptable computing time. Hence, the principled approach is a significant improvement to existing methods that are used to draw quasi-orders uniformly at random but cannot cope with reasonably large item sets.

## 1. Introduction

We begin with motivational considerations. We address why discrete modeling with quasi-orders is useful and why we need to sample quasi-orders we want to be representative. In addition, this section gives an overview of the main contributions and organization of this paper.

### 1.1. Why discrete order structures are important

A *quasi-order* on a set, for instance, of educational or psychological test or questionnaire items, is any binary relation that is reflexive and transitive. Relational dependencies or discrete order structures such as the quasi-orders can model the dependencies of mastery or precedence relations among the problems of an achievement test or the statements of an attitude questionnaire. The general idea is to represent any empirically plausible dependency of the type “*The mastery of problem y implies the mastery of problem x*” between the questions *x* and *y* of the test *I* as the item pair *x* ≤ *y* of a quasi-order ≤ on *I*. This quasi-order structure imposed on the test can be employed to design efficient test administration procedures. One can mimic the adaptive approach of a teacher, for instance, when the teacher's experience and knowledge about the prerequisite relations between the problems are used to avoid asking a student questions that are either too easy or too difficult. The most pertinent protagonist of this idea is the *theory of knowledge or learning spaces* (Doignon and Falmagne, 1985, 1999; Falmagne and Doignon, 2011; Falmagne et al., 2013). In this theory, discrete mathematical concepts, including the quasi-orders, have played an important role. They have been employed for the adaptive modeling, assessment, and training of knowledge, competence, and learning dynamics in human (e.g., student) populations. More generally, orders may be deemed a pivotal contribution to the behavioral and social sciences, amongst others. For a thorough motivation of orders and knowledge or learning space theory, including further references (see Schrepp and Ünlü, 2015).

### 1.2. Why representative quasi-order samples are important

Methods that reconstruct quasi-orders from empirical data are computational. Examples are the algorithms of *item tree analysis* (van Leeuwe, 1974; Schrepp, 1999; Sargin and Ünlü, 2009). For applications of item tree analysis to real datasets in knowledge or learning space theory, (see also Schrepp, 2002, 2003, 2006; Ünlü and Sargin, 2010). Computational methods of this sort have been developed, evaluated, and compared predominantly based on extensive simulation studies. It is worth mentioning that simulation is *the* key methodology relied on in this field, as the objective as well as systematic approach to studying these computer-oriented data mining techniques. The design of the conducted simulation studies critically depends on large samples of randomly generated quasi-orders used at their basis. Why? Each quasi-order of the sample is posited to represent the true relational dependencies that a tested mining algorithm has to reconstruct from simulated data, so one wants to ensure that no interesting quasi-order has been missed. All of the algorithms depend on the underlying quasi-order structure. For some structural types, it may be easier to detect the correct dependencies based on a dataset compared with others, and this may vary across the methods or with different datasets. Moreover, in practical contexts, the structure of the true quasi-order is typically unknown. These considerations warrant the importance of simulation studies and of controlling in these studies for the dependency on quasi-order structure.

If we do not want to exclude quasi-orders a priori from consideration, which is generally not ideal, a natural solution is to evaluate and compare the performance of the mining algorithms in the set of all possible quasi-orders. However, considering all of the quasi-orders in a simulation study is not feasible in general. A sample is needed. Once again, a natural choice is to give each quasi-order on the item set the same chance of being included in the simulation study. This will produce the least-biased results when generalizing the findings obtained from the simulation study to the population of all possible quasi-orders on the item set. Thus, it is essential for us to base any simulation study that aims to investigate the performance of such data mining techniques in a meaningful and reliable manner on *representative* quasi-order samples.

**Definition 1**. *In the sequel, the* representativeness *of a random sample of quasi-orders means that each quasi-order on the item set has the same probability of being selected as part of the sample*.

Why sampling quasi-orders is necessary for us was also concretized in Ünlü and Schrepp (2015). In their study, the importance of representative sampling of quasi-orders and the biases and errors induced by non-representative samples were clearly evidenced. The representativeness of the quasi-orders employed in extensive simulation studies was seen to be an important requirement for the sound comparison of such exploratory data analysis methods as item tree analysis. In particular, Ünlü and Schrepp (2015) found that utilizing non-representative quasi-order samples yielded biased simulative assessment results with regard to the recovery and coverage qualities associated with the existing item tree analysis algorithms. For further motivation of representative random quasi-orders (see also Schrepp and Ünlü, 2015, Section Introduction).

### 1.3. Content and structure of this work

Schrepp and Ünlü (2015) introduced an inductive algorithm, which represents the state-of-affairs sampling technique for quasi-orders. In this procedure, trace quasi-orders of lower dimension *l* are extended, uniformly at random, to dimension *l* + 1. This construction step is described later in detail. It constitutes one of the two inductive components of the proposed procedure. These random extensions are checked for transitivity. Transitive extensions are retained. Non-transitive relations are rejected without further analysis. This algorithm improves on two direct methods for drawing representative random quasi-orders (for details, see Schrepp and Ünlü, 2015). However, when the number of items *n* increases, all of these procedures become computationally too intensive, particularly because the proportion of extensions representing quasi-orders decreases very quickly with *n*.

We introduce a constructive procedure that in a second inductive step corrects the extensions that violate the transitivity property. Thus, on *all* trials of the new procedure, quasi-orders are obtained. Correcting for transitivity in a combinatorial manner, this randomized doubly inductive procedure is biased. However, bias correction is possible. Three algorithms are proposed. A truly representative variant, termed *absolute rejection method*, outright rejects the randomly generated quasi-orders based on the penalizing weights that can be computed using the inductive correction procedure. Here, the *penalizing weight* corresponding to a random quasi-order is the number of possible uniform extensions that, when being corrected according to the algorithm, do yield the quasi-order under reference. The second and third variants, respectively termed *simple resampling method* and *stratified resampling method*, apply proportional weighting based on the procedural bias correction factors. These methods take resamples from the constructed sample as if it were the population. The simple resampling method operates on the quasi-orders directly as the units being weighted and resampled. With the stratified resampling method, the quasi-orders of the sample are divided into strata defined by those weights before resampling. The strata are the units being weighted and resampled, and simple random sampling is applied within each drawn stratum to obtain a quasi-order sample. The two resampling-based methods are the recommended procedures. In extensive simulation studies, we will see that these algorithms are efficient and feasible for reasonably large item sets while providing close to representative random quasi-order samples.

This paper is organized as follows. In Section 2, we describe the methods currently available for sampling quasi-orders, including the pertinent inductive uniform extension approach by Schrepp and Ünlü (2015). In Section 3, we introduce the discrete doubly inductive procedure for the construction of potentially all quasi-orders on a finite item set. In Section 4, the doubly inductive procedure is randomized, thereby yielding a probabilistic procedure for quasi-order sampling. The sampling biases induced in the process of randomization are addressed, and the corresponding bias correction factors are derived. In Section 5, we propose the three algorithms for bias correction, the absolute rejection method, the simple resampling method, and the stratified resampling method. Section 6 reports the simulation results obtained for these sampling techniques. In Section 7, we summarize our findings, and we conclude with final remarks and suggestions for further research.

## 2. State-of-the-art sampling techniques

### 2.1. Flexible but non-representative *ad hoc* strategies

We present two example strategies of this sort that have been published in the literature. Because these methods are *ad hoc*, modifications or alternative procedures are easily possible. *Ad hoc* strategies are flexible and quick to compute. However, they generally lack representativeness of the generated sets of quasi-orders. For this class of procedures, it seems to be very complicated to address the issue of representativeness on a principled theoretical basis, if it can be addressed at all. Nonetheless, samples obtained from these techniques may approximate true distributions reasonably well by adjusting their parameters fittingly.

One method is based on the normal distribution, the other on the uniform distribution. Both come in two variants, absolute and averaged. Let *I* be an item set of size |*I*| = *n*.

1. Start with the diagonal relation on *I* consisting of all reflexive item pairs (*i, i*) with *i* ∈ *I*.
2. Let δ ~ *N*(μ, σ) for the normal method or δ ~ *U*(0, *b*) for the uniform method. The parameters μ (mean), σ (standard deviation), and *b* (upper interval bound) are specified in such a way that the realization δ constitutes a probability value between 0 and 1.
  Example specifications in Sargin and Ünlü (2009) are μ = 0.16 and σ = 0.06, with the additional boundary restrictions that δ values < 0 or > 0.3 are set to 0 or 0.3, respectively. For the uniform method (Schrepp, 1999), *b* can be set to 0.4 or 1, for instance.
3. For any non-reflexive item pair, add that pair to the diagonal relation with probability δ (or discard it with probability 1 − δ). This yields a binary relation R, which is reflexive.
4. To satisfy transitivity, take the transitive closure of R. (The *transitive closure* of a binary relation R on an item set *I* is the smallest binary relation on *I* that contains R and is transitive. Note that the transitive closure always exists for any binary relation.) The resulting binary relation is the random quasi-order obtained according to the *ad hoc* strategy.
5. In the absolute variant, for each random δ, only one random quasi-order is drawn. In the averaged variant, for each random δ, multiple random quasi-orders are generated and jointly used in the analyses.

As shown in Ünlü and Schrepp (2015), these *ad hoc* random processes yield non-representative quasi-order samples. In decreasing order of representativeness were the averaged followed by the absolute normal variants, whereas both variants of the uniform method produced the worst results with random samples of overly represented large quasi-orders.

### 2.2. Representative but infeasible direct methods

Two direct or natural sampling techniques that do yield representative random quasi-orders are census-like and entry-wise uniform sampling.

In *census-like uniform sampling*, all possible quasi-orders on a small-sized item set are constructed and known. The quasi-orders are randomly chosen from an accessible population. However, constructing, storing, and uniformly sampling from a known population only works for a small item number *n*. The total (labeled) quasi-order counts increase very rapidly (Brinkmann and McKay, 2002, 2005; Pfeiffer, 2004). For example, the counts are 9, 535, 241/642, 779, 354/63, 260, 289, 423/8, 977, 053, 873, 043 for 7/8/9/10 items, respectively. In Ünlü and Schrepp (2015), the census-like sampling approach was demonstrated with six items, where we have a total of 209, 527 quasi-orders in the population. In this method, each draw, if feasible, is a quasi-order, although the equal sampling probability for each quasi-order may be very small.

*Entry-wise uniform sampling* uses the relational (or adjacency) matrix representation of a quasi-order (defined below). For reflexivity, the diagonal entries are set to 1 beforehand. Each of the remaining entries of the relational matrix are randomly filled with equal probability 1/2: 1 (in relation) or 0 (not in relation). The resulting random reflexive relation is retained if it satisfies transitivity. Otherwise, the relation is rejected without further analysis.^1^ This procedure also becomes infeasible in *n* (Schrepp and Ünlü, 2015). The probability of selecting any of the reflexive and, in particular, transitive relations is the same, 0.5^*n*(*n* − 1)^. The proportion of quasi-orders among all reflexive relations very rapidly decreases with increasing item number *n*. There are 2^*n*(*n* − 1)^ reflexive relations, and for 6 ≤ *n* ≤ 10, the proportions are 1.95·10^−4^, 2.17·10^−6^, 8.92·10^−9^, 1.34·10^−11^, and 7.25·10^−15^, respectively. This very small proportion gives the probability for a draw to result in the set of all quasi-orders on *n* items, denoted by $Q_{n}$. Thus, draws under entry-wise uniform sampling are almost exclusively reflexive relations that do not satisfy the transitivity property. This is true especially in realistic contexts with larger item numbers. However, given a draw that occurs in $Q_{n}$, the probability for any quasi-order of being selected is the same, $1/|Q_{n}|$.

### 2.3. Inductive uniform extension approach by Schrepp and Ünlü (2015)

The direct procedures are theoretically representative but practically infeasible. The *ad hoc* procedures are practically feasible but theoretically not representative. The inductive uniform extension approach by Schrepp and Ünlü (2015) is a good compromise in this regard. It relies on the same idea as the entry-wise uniform sampling but at a more informational level of matrix structures. For these matrix structures, the proportion of quasi-orders becomes sparse only for higher item numbers. Thus, the inductive procedure improves on the feasibility of the entry-wise uniform sampling method for larger values of *n*. It can be shown that samples generated under this approach remain representative. For details (see Schrepp and Ünlü, 2015).

The inductive uniform extension technique is essential. It constitutes one of the two inductive components of the proposed randomized doubly inductive procedure. To describe this method, we introduce the required notation. Let *R* ⊂ *I* × *I* be a binary relation on the item set *I* = {1, 2, …, *n*}. A pair (*i, j*) ∈ *R* is also denoted by *iRj* for *i, j* ∈ *I*. The *relational* or *adjacency matrix r*_*R*_ of *R* is the binary matrix (rij)i=1,…,n,j=1,…,n (indexing omitted subsequently) defined by *r*_*ij*_ = 1 if *iRj*, and 0 otherwise.

In this notation, “*R* is reflexive” means *r*_*ii*_ = 1 for all *i* = 1, …, *n*. The transitivity of *R* states that for all 1 ≤ *i, j, k* ≤ *n*, if *r*_*ij*_ = 1 and *r*_*jk*_ = 1, then *r*_*ik*_ = 1. Moreover, the entry-wise uniform sampling can be recapped: *r*_*ii*_ : = 1 for all *i* ∈ *I*, and *r*_*ij*_ ~ _*iid*_ *Bernoulli*(1/2) for all *i, j* ∈ *I* with *i* ≠ *j*. Here, *Bernoulli*(1/2) is the Bernoulli distribution with success (i.e., *r*_*ij*_ = 1) probability *p* = 1/2, and *iid* stands for “independent and identically distributed” (subsequently being omitted). In the entry-wise uniform sampling, all off-diagonal entries of the relational matrix are randomly filled. Exemplified with *n* = 3 items, these entries are marked:

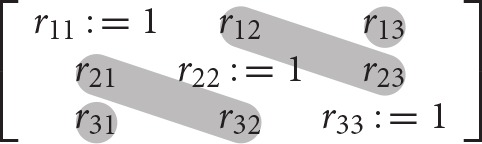



and *r*_12_, *r*_13_, *r*_21_, *r*_23_, *r*_31_, *r*_32_ ~ *Bernoulli*(1/2).

Let *R* be a *trace quasi-order* on the items 1, …, *l*. In the inductive uniform extension approach, we construct a random reflexive relation on the items 1, …, *l, l* + 1, which extends the relational matrix *r*_*R*_ of *R* with a new (*l* + 1)th row and (*l* + 1)th column, retaining the original values of *r*_*R*_. The new entries are randomly filled (except for the diagonal element, which is set to 1). Exemplified with *l* = 2, for a *trace adjacency matrix r*_*R*_ of dimension 2 × 2, the randomly filled entries are marked:

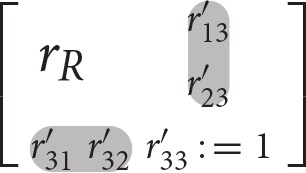



and $r_{13}^{\prime },r_{23}^{\prime },r_{31}^{\prime },r_{32}^{\prime }\sim Bernoulli\left(1/2\right)$.

More generally, we use a random variable formulation. Let (*r*_*ij*_) be a *trace reflexive matrix* of independent random variables *r*_*ij*_ ~ *Bernoulli*(*p*_*ij*_), with success probabilities *p*_*ij*_ of either 0, 1, or 1/2, and with *p*_*ii*_ = 1 for all *i* = 1, …, *l*. Any realization of this matrix random vector defines the relational (or adjacency) matrix of a random reflexive relation on *I* = {1, …, *l*}. A *random variable reflexive extension* of this trace matrix random vector is the matrix $\left(r_{ij}^{\prime }\right)$ of random variables $r_{ij}^{\prime }:=r_{ij}$ for all *i, j* = 1, …, *l* (extension), of $r_{\left(l+1\right)j}^{\prime }\sim Bernoulli\left(p_{\left(l+1\right)j}\right)$ for *j* = 1, …, *l* + 1 with *p*_(*l* + 1)*j*_ = 1 for *j* = *l* + 1 (reflexivity) and 1/2 otherwise, and of $r_{i\left(l+1\right)}^{\prime }\sim Bernoulli\left(p_{i\left(l+1\right)}\right)$ for *i* = 1, …, *l* + 1 with *p*_*i*(*l* + 1)_ = 1/2 for all *i* ≠ *l* + 1. Any realization of this random variable reflexive extension (on *l* + 1 items) that coincides with a realization of the trace matrix random vector (on *l* items) is called a *random reflexive extension* of this *trace reflexive relation*.

The method proposed by Schrepp and Ünlü (2015) is *inductive* and relies on this notion of a random reflexive extension.

**Anchoring**. The inductive procedure starts with a representative sample of quasi-orders on a sufficiently small number of items, *l*. This may include the complete inventory of all possible quasi-orders.
  For example, the procedure can be anchored by using the set of all 355 (labeled) quasi-orders on *l* = 4 items, or with a simple random sample of 1000 quasi-orders for *l* = 6 items.
**Inductive step**. Suppose we have a representative sample of quasi-orders on *n* ≥ *l* items, denoted by Q(*n*). (Note that $Q\left(n\right)\subset Q_{n}$.) For each quasi-order in Q(*n*), we compute a pre-specified number *z* of random reflexive extensions of the quasi-order. These extensions are checked for transitivity. Non-transitive extensions are excluded without further analysis. The transitive extensions are added to a new collection of quasi-orders on *n* + 1 items, Q(*n* + 1).
  Modifications are possible. Duplicates can be removed from Q(*n* + 1), depending on the envisaged application. An intermediate-step Q(*n* + 1) can be reduced to a simple random sample of feasibly limited size if the inductive construction is repeated several times to run from small to larger item numbers.

Schrepp and Ünlü (2015) showed that this procedure theoretically yields representative samples. Their study also investigated the quality of the inductive uniform extension approach in simulation. On up to 15 items, this method created representative quasi-order samples within acceptable computing time. This procedure improves on the two direct methods (Section 2.2). However, with more items, it too becomes computationally intensive. The randomized doubly inductive procedure described in the following sections significantly improves on the efficiency and feasibility of this method.

## 3. Doubly inductive procedure for quasi-order construction

The driving force for the new procedure is to develop a discrete combinatorial algorithm for the construction of, in principle, all quasi-orders on a given item set (the *doubly inductive* component). This algorithm then can be obtained probabilistically by randomization in individual construction steps (the *randomized* component), eventually yielding a random process for quasi-order sampling.

### 3.1. Description of the deterministic construction procedure

The discrete construction procedure can be termed *doubly inductive* in the following sense. The *outer level*, or *Level 2*, parallels the inductive uniform extension method (UEM) by Schrepp and Ünlü (2015). The trace quasi-orders of lower dimensions, *l* < *n*, are successively extended in each step by one more item, *l* + 1, to eventually yield final quasi-orders on *n* items. The *inner level*, or *Level 1*, is nested within each unit (i.e., intermediate *l*-dimensional trace quasi-order) at the higher Level 2. The entries of the added (*l* + 1)th column and (*l* + 1)th row of the relational matrix are filled, again inductively, according to a specific procedure we call top-down–right-left inductive discrete extension.

**Figure 1:**
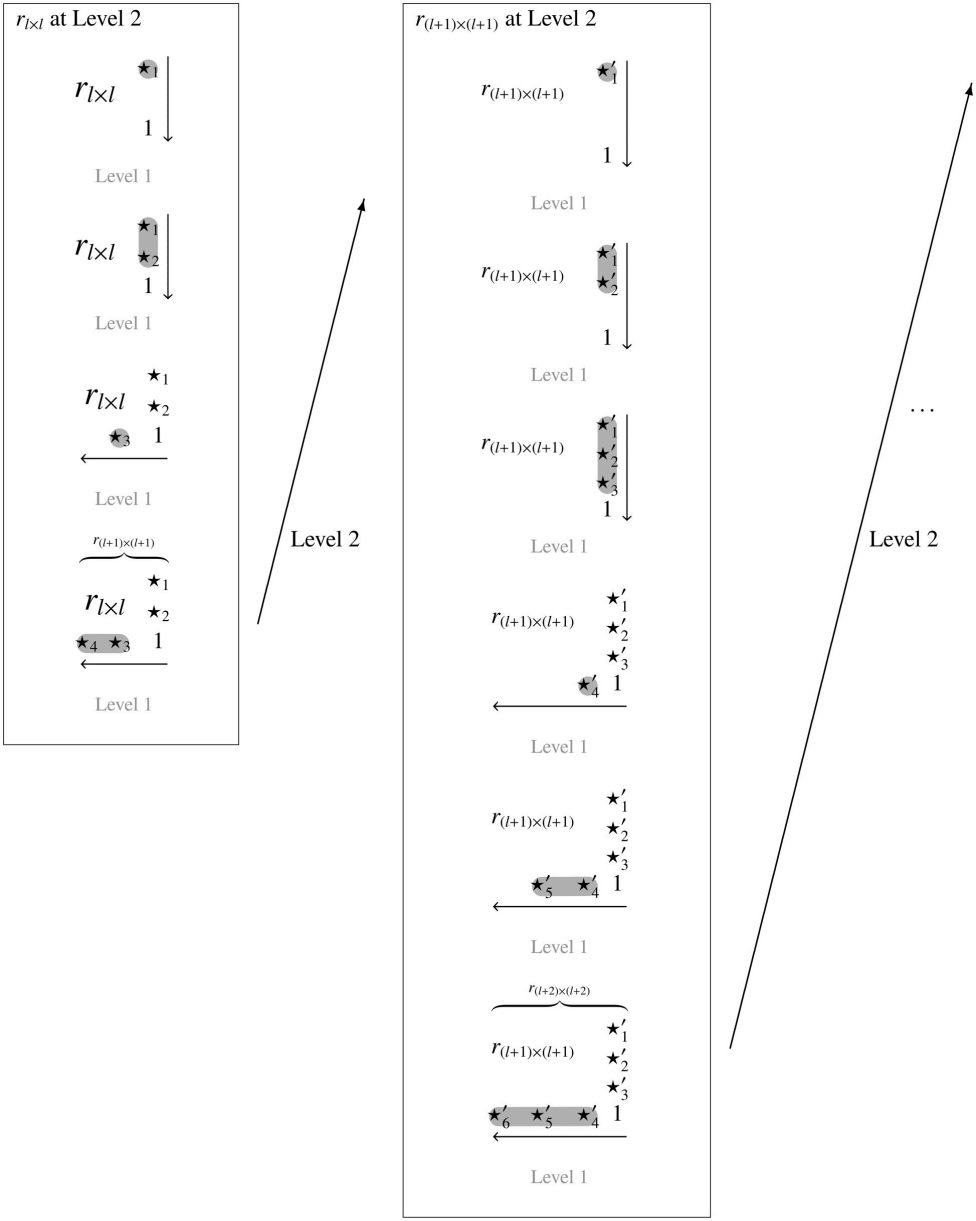
**The doubly inductive construction procedure exemplified with ***l*** = 2**. For one Level 2 inductive step leading from *l* to *l* + 1, and with four and six Level 1 inductive steps within the Level 2 units or trace quasi-orders (in relational matrix notation), *r*_*l* × *l*_ and *r*_(*l* + 1) × (*l* + 1)_, respectively. This leads to discrete reflexive extensions of *r*_*l* × *l*_ on three items and of *r*_(*l* + 1) × (*l* + 1)_ on four items. The symbols ⋆_*i*_ and ${⋆}_{i}^{\prime }$ denote the entries of the adjacency matrices that are deterministically filled with 0's and 1's (random fillings follow later) according to the top-down–right-left inductive extension.

This strategy starts from a given trace quasi-order on the items 1, …, *l* and extends it, in doubly inductive manner, to a quasi-order on the items 1, …, *l, l* + 1, …, *n*. This approach is conceptually depicted by Figure 1.

Throughout, we use the adjacency matrix notation. Let *n* : = {1, …, *n*}. The set of all quasi-orders on *n* is $Q_{n}$. The introduced procedure allows one to construct from $Q_{n}$ the set of all quasi-orders on *n* + 1, *Q*_*n* + 1_. For $r_{n}=\left(r_{ij}\right)\in Q_{n}$, let $r_{n}^{◇}\;$: = {*r*_*n* + 1_ ∈ *Q*_*n* + 1_ : *r*_*n* + 1_⋂*n* × *n* = *r*_*n*_}. That is, $r_{n}^{◇}$ is the *parent family* of *r*_*n*_ of quasi-orders or extensions *r*_*n* + 1_ on *n* + 1 that coincide with *r*_*n*_ when restricted to *n* ⊂ *n* + 1. It holds that

$$Q_{n+1}=\underset{r_{n}\in Q_{n}}{\sum }r_{n}^{◇},$$

in the sense that $\left\{r_{n}^{◇}:r_{n}\in Q_{n}\right\}$ forms a partition of $Q_{n+1}$. Thus, the target quasi-orders in $Q_{n+1}$ can be generated by constructing for any trace quasi-order $r_{n}\in Q_{n}$ the corresponding parent family $r_{n}^{◇}$. This can be achieved as follows.

Let the additional (*n* + 1)th column and (*n* + 1)th row of any extension $r_{n+1}\in r_{n}^{◇}$ of $r_{n}\in Q_{n}$ be denoted by *r*_1, *n* + 1_, *r*_2, *n* + 1_, …, *r*_*n, n* + 1_ and *r*_*n* + 1, *n*_, *r*_*n* + 1, *n* − 1_, …, *r*_*n* + 1, 1_, which are listed in the order in which they are filled. Note that all other entries of *r*_*n* + 1_ are known. More precisely, *r*_*n* + 1, *n* + 1_: = 1, and the entries related to *n* are inherited from *r*_*n*_. Thus, the construction of $r_{n}^{◇}$ means constructing all 2*n*-dimensional binary vectors

$$\begin{array}{l} x=\left(r_{1,n+1},r_{2,n+1},\ldots ,r_{n,n+1},r_{n+1,n},r_{n+1,n-1},\ldots ,r_{n+1,1}\right),\\ x\in {\left\{0,1\right\}}^{2n} \end{array}$$

such that *r*_*n*+1_(*x*), that is, the matrix *r*_*n*_ extended with these fillings, satisfies the transitivity property. Instead of filling these entries all at once and then testing for transitivity overall in the full matrix, the construction is inductive. A next step of the construction is built based on the construction steps preceding it.

Here is the description of the *top-down–right-left inductive discrete extension* procedure. We will verify that this procedure leads to a construction of the parent family $r_{n}^{◇}$ (Proposition 2).

**Filling the column *n*** + **1 such that transitivity holds (***top - down*** component):**

The order of filling the column entries is *r*_1, *n* + 1_, followed by *r*_2, *n* + 1_, …, and finally, *r*_*n, n* + 1_ is filled (see Figure 1).

**Anchoring**. The first entry *r*_1, *n* + 1_ can be set to any of the values 0 and 1. No violation of transitivity occurs in either case in the sense of the two conditions required when filling the next entries *r*_*k, n* + 1_, *k* = 2, …, *n* (detailed below in the inductive step).
  For each of the admissible values *r*_1, *n* + 1_ : = 0 and 1, all of the subsequent construction steps are carried out.
**Inductive step**. Suppose the *K* entries *r*_*k, n* + 1_ for 1 ≤ *k* ≤ *K* < *n* have been filled with 1's or 0's such that the following two *transitivity conditions* are satisfied, respectively:^2^

**Condition *C_1_*(*k*), when *r_k,n+1_* := *1***. For all *i* ∈ {1, …, *k* − 1}, it holds that *r*_*i, k*_ = 0 or *r*_*i, n* + 1_ = 1 (inclusive “or”).
**Condition *C_2_*(*k*), when *r_k,n+1_* := *0***. For all *i* ∈ {1, …, *k* − 1}, it holds that *r*_*k, i*_ = 0 or *r*_*i, n* + 1_ = 0.
  For the construction procedure to yield the whole set $r_{n}^{◇}$, all of the admissible values for any of the entries *must* be combined with one another.
  For the (*k* + 1)th entry *r*_*k* + 1, *n* + 1_, the inductive step, 1 or 0 must be assigned to *r*_*k* + 1, *n* + 1_, if they are admissible, that is, if *C*_1_(*k* + 1) or *C*_2_(*k* + 1) are satisfied, respectively. According to Proposition 2, at least one of the two conditions necessarily holds true.
  For each of the admissible values for *r*_*k* + 1, *n* + 1_, all of the subsequent construction steps are carried out.
  The inductive step is repeated until *K* = *n* and the column *n* + 1 is fully specified. We denote with $S_{1}=S_{1}\left(r_{n}\right)$ the set of all possible specifications of admissible values for the (*n* + 1)th column, which depends on the trace quasi-order $r_{n}\in Q_{n}$.

An example may help to illustrate this top-down construction component. On *n* = 2 items, consider the trace quasi-order

$$\left[\begin{array}{cc} 1 & 1\\ 1 & 1 \end{array}\right].$$

The entry *r*_1, 3_ of the added third column can be set to any of the admissible values 0 and 1,

$$\begin{array}{l} \left[\begin{array}{ccc} 1 & 1 & \begin{array}{c} 0 \end{array}\\ 1 & 1 & \\ & & 1 \end{array}\right]\;\left[\begin{array}{ccc} 1 & 1 & \begin{array}{c} 1 \end{array}\\ 1 & 1 & \\ & & 1 \end{array}\right]. \end{array}$$

Filling the entry *r*_2, 3_ yields the following possible patterns

$$\left[\begin{array}{ccc} 1 & 1 & 0\\ 1 & 1 & \begin{array}{c} 0 \end{array}\\ & & 1 \end{array}\right]\;\left[\begin{array}{ccc} 1 & 1 & 0\\ 1 & 1 & \begin{array}{c} 1 \end{array}\\ & & 1 \end{array}\right]\;\left[\begin{array}{ccc} 1 & 1 & 1\\ 1 & 1 & \begin{array}{c} 0 \end{array}\\ & & 1 \end{array}\right]\;\left[\begin{array}{ccc} 1 & 1 & 1\\ 1 & 1 & \begin{array}{c} 1 \end{array}\\ & & 1 \end{array}\right],$$

where the values not admissible for this entry are highlighted in red. The two patterns containing inadmissible values are rejected in subsequent construction steps.

For any resulting and thus subsequently given values

$$c=\left(r_{1,n+1},r_{2,n+1},\ldots ,r_{n,n+1}\right)\in S_{1},$$

the procedure continues to fill the entries of the (*n* + 1)th row.

**Filling the row *n*** + **1 such that transitivity holds (*right* - *left* component):**

The order of filling the row is *r*_*n* + 1, *n*_, *r*_*n* + 1, *n* − 1_, …, *r*_*n* + 1, 1_ (see Figure 1). In this case, the value 1 is called *admissible* for an entry *r*_*n* + 1, *k*_ (1 ≤ *k* ≤ *n*) if the following two *transitivity conditions* are satisfied:^3^

**Condition *R_1a_*(*k*), when *r_n+1,k_* := *1***. For all *i* ∈ {1, …, *n*} \ {*k*}, it holds that *r*_*i, k*_ = 1 or *r*_*i, n* + 1_ = 0.
**Condition *R_1b_*(*k*), when *r_n+1,k_* := *1***. For all *i* ∈ {*k* + 1, …, *n*}, it holds that *r*_*k, i*_ = 0 or *r*_*n* + 1, *i*_ = 1.

The value 0 is *admissible* for an entry *r*_*n* + 1, *k*_ (1 ≤ *k* ≤ *n*) if the following *transitivity condition* is fulfilled:

**Condition *R_2_*(*k*), when *r_n+1,k_* := *0***. For all *i* ∈ {*k* + 1, …, *n*}, it holds that *r*_*i, k*_ = 0 or *r*_*n* + 1, *i*_ = 0.

All of the admissible values for any of the entries *must* be combined with one another for the construction procedure to yield every element of $r_{n}^{◇}$.

**Anchoring**. The first entry *r*_*n* + 1, *n*_ can be set to 0, since *R*_2_(*k* = *n*) is trivially satisfied (independent of any given $c\in S_{1}$). The value 1 is admissible for *r*_*n* + 1, *n*_ if condition *R*_1*a*_(*n*) is satisfied [condition *R*_1*b*_(*n*) holds true trivially]. For each of the admissible values for *r*_*n* + 1, *n*_, all of the subsequent construction steps are carried out.
**Inductive step**. Suppose the *K* entries *r*_*n* + 1, *k*_ (1 < *n* − *k* + 1 ≤ *k* ≤ *n*) have been filled with 1's or 0's such that the conditions *R*_1*a*_(*k*) and *R*_1*b*_(*k*) or *R*_2_(*k*) are satisfied, respectively.
  For the (*k* + 1)th entry *r*_*n* + 1, *n* − *K*_, the inductive step, 1 or 0 must be assigned to *r*_*n* + 1, *n* − *K*_, if they are admissible, that is, if the conditions *R*_1*a*_(*n* − *K*) and *R*_1*b*_(*n* − *K*) or *R*_2_(*n* − *K*) are satisfied, respectively. According to Proposition 2, at least one of the conditions *R*_1*a*_(*n* − *K*) and *R*_1*b*_(*n* − *K*) or *R*_2_(*n* − *K*) necessarily holds true.
  For each of the admissible values for *r*_*n* + 1, *n* − *K*_, all of the subsequent construction steps are carried out.
  The inductive step is repeated until *K* = *n* and the row *n* + 1 is fully specified. We denote with $S_{2}=S_{2}\left(c,r_{n}\right)$ the set of all possible specifications of admissible values for the (*n* + 1)th row, which depends on the vector $c=\left(r_{1,n+1},r_{2,n+1},\ldots ,r_{n,n+1}\right)\in S_{1}$ and trace quasi-order $r_{n}\in Q_{n}$.

In the above example, the patterns to be further filled are

$$\left[\begin{array}{ccc} 1 & 1 & 0\\ 1 & 1 & 0\\ & & 1 \end{array}\right]\;\left[\begin{array}{ccc} 1 & 1 & 1\\ 1 & 1 & 1\\ & & 1 \end{array}\right].$$

Filling the entry *r*_3, 2_ gives

$$\left[\begin{array}{ccc} 1 & 1 & 0\\ 1 & 1 & 0\\ & \begin{array}{c} 0 \end{array} & 1 \end{array}\right]\;\left[\begin{array}{ccc} 1 & 1 & 0\\ 1 & 1 & 0\\ & \begin{array}{c} 1 \end{array} & 1 \end{array}\right]\;\left[\begin{array}{ccc} 1 & 1 & 1\\ 1 & 1 & 1\\ & \begin{array}{c} 0 \end{array} & 1 \end{array}\right]\;\left[\begin{array}{ccc} 1 & 1 & 1\\ 1 & 1 & 1\\ & \begin{array}{c} 1 \end{array} & 1 \end{array}\right].$$

All of the possible patterns contain admissible values. The entry *r*_3, 1_ must be filled for each of these patterns, yielding

$$\left[\begin{array}{ccc} 1 & 1 & 0\\ 1 & 1 & 0\\ \begin{array}{c} 0 \end{array} & 0 & 1 \end{array}\right]\;\left[\begin{array}{ccc} 1 & 1 & 0\\ 1 & 1 & 0\\ \begin{array}{c} 0 \end{array} & 1 & 1 \end{array}\right]\;\left[\begin{array}{ccc} 1 & 1 & 1\\ 1 & 1 & 1\\ \begin{array}{c} 0 \end{array} & 0 & 1 \end{array}\right]\;\left[\begin{array}{ccc} 1 & 1 & 1\\ 1 & 1 & 1\\ \begin{array}{c} 0 \end{array} & 1 & 1 \end{array}\right]$$

and

$$\left[\begin{array}{ccc} 1 & 1 & 0\\ 1 & 1 & 0\\ \begin{array}{c} 1 \end{array} & 0 & 1 \end{array}\right]\;\left[\begin{array}{ccc} 1 & 1 & 0\\ 1 & 1 & 0\\ \begin{array}{c} 1 \end{array} & 1 & 1 \end{array}\right]\;\left[\begin{array}{ccc} 1 & 1 & 1\\ 1 & 1 & 1\\ \begin{array}{c} 1 \end{array} & 0 & 1 \end{array}\right]\;\left[\begin{array}{ccc} 1 & 1 & 1\\ 1 & 1 & 1\\ \begin{array}{c} 1 \end{array} & 1 & 1 \end{array}\right].$$

Inadmissible values for this entry are shown in red and the corresponding matrices do violate the transitivity conditions. Thus, the parent family of the quasi-order

$$r_{2}=\left[\begin{array}{cc} 1 & 1\\ 1 & 1 \end{array}\right]$$

of all reflexive and transitive extensions on 3 that coincide with *r*_2_ when restricted to 2 ⊂ 3 is

$$r_{2}^{⋄}=\left\{\left[\begin{array}{ccc} 1 & 1 & 0\\ 1 & 1 & 0\\ 0 & 0 & 1 \end{array}\right]\;,\;\left[\begin{array}{ccc} 1 & 1 & 0\\ 1 & 1 & 0\\ 1 & 1 & 1 \end{array}\right]\;,\;\left[\begin{array}{ccc} 1 & 1 & 1\\ 1 & 1 & 1\\ 0 & 0 & 1 \end{array}\right]\;,\;\left[\begin{array}{ccc} 1 & 1 & 1\\ 1 & 1 & 1\\ 1 & 1 & 1 \end{array}\right]\right\}.$$

### 3.2. Properties of the doubly inductive procedure

We discuss a few important properties of this construction procedure. From the example above, we can see that for any position that is filled, one or both of the values 0 or 1 is admissible. For instance, in the construction step

$$\left[\begin{array}{ccc} 1 & 1 & 0\\ 1 & 1 & 0\\ & & 1 \end{array}\right]\to \left[\begin{array}{ccc} 1 & 1 & 0\\ 1 & 1 & 0\\ & \begin{array}{c} 0 \end{array} & 1 \end{array}\right]\;,\;\left[\begin{array}{ccc} 1 & 1 & 0\\ 1 & 1 & 0\\ & \begin{array}{c} 1 \end{array} & 1 \end{array}\right],$$

both 0 and 1 are admissible values for the entry *r*_3, 2_. In a subsequent step of filling the entry *r*_3, 1_,

$$\left[\begin{array}{ccc} 1 & 1 & 0\\ 1 & 1 & 0\\ & 0 & 1 \end{array}\right]\to \left[\begin{array}{ccc} 1 & 1 & 0\\ 1 & 1 & 0\\ \begin{array}{c} 0 \end{array} & 0 & 1 \end{array}\right],$$

the value 0 is the only admissible. Moreover, surveying the known population of all quasi-orders on a set of three items, we have verified in the example that the extensions constructed according to the procedure are exactly those quasi-orders in that population which have the initial quasi-order *r*_2_ as the trace. In the example, the corresponding parent family $r_{2}^{◇}$ has been constructed and consists of four quasi-orders.

The afore mentioned properties are not specific to the example and can be proven in the general case.

**Proposition 2**. *Under the aforementioned prerequisites and notation, we have:*

1. *In any step of the construction procedure, that is, for any of the entries r*_1, *n* + 1_, *r*_2, *n* + 1_, …, *r*_*n, n* + 1_, *r*_*n* + 1, *n*_, *r*_*n* + 1, *n* − 1_, …, *r*_*n* + 1, 1_*, at least one of the values* 1 *or* 0 *is always admissible for the position that is filled. More precisely:*
  - *In the case of filling column n* + 1*, for any position k* = 1, …, *n, at least one of the conditions C*_1_(*k*) *or C*_2_(*k*) *is satisfied*.
  - *In the case of filling row n* + 1*, for any position k* = 1, …, *n, the conditions R*_1*a*_(*k*) *and R*_1*b*_(*k*) *are satisfied or the condition R*_2_(*k*) *is fulfilled*.
2. *The set of all relational matrices resulting from this construction is equal to the parent family* $r_{n}^{◇}$ *of* $r_{n}\in Q_{n}$*. More precisely:*
  $$\begin{array}{l} T\;:=\left\{r_{n+1}\left(x\right):x=\left(c_{1},c_{2}\right),c_{1}\in S_{1}\left(r_{n}\right),and\;c_{2}\in S_{2}\left(c_{1},r_{n}\right)\right\}=r_{n}^{◇}, \end{array}$$
  *where r*_*n* + 1_(*x*) *is the matrix r*_*n*_ *extended with the fillings in x as the* (*n* + 1)*th column and* (*n* + 1)*th row added to r*_*n*_.

*Proof*. 1, a. Assume that *k* > 1 is the smallest position such that both conditions *C*_1_(*k*) and *C*_2_(*k*) are violated. Then, there exist an *i*_1_ ≤ *k* − 1 with *r*_*i*_1_, *k*_ = 1 and *r*_*i*_1_, *n* + 1_ = 0, and an *i*_2_ ≤ *k* − 1 with *r*_*k*,*i*_2__ = 1 and *r*_*i*_2_, *n* + 1_ = 1. If *i*_1_ = *i*_2_, the contradiction is 0 = *r*_*i*_1_, *n* + 1_ = *r*_*i*_2_, *n* + 1_ = 1. Let *i*_1_ ≠ *i*_2_. Since *r*_*n*_ is a quasi-order on *n*, we have *r*_*i*_1_, *i*_2__ = 1. If *i*_1_ < *i*_2_, since *k* is the smallest such critical position and *r*_*i*_2_, *n* + 1_ = 1, the condition *C*_1_(*i*_2_) is satisfied. Since *r*_*i*_1_, *n* + 1_ = 0, it follows 0 = *r*_*i*_1_, *i*_2__ = 1. If *i*_2_ < *i*_1_, because *r*_*i*_1_, *n* + 1_ = 0, the condition *C*_2_(*i*_1_) is fulfilled. Since *r*_*i*_2_, *n* + 1_ = 1, the resulting contradiction is 0 = *r*_*i*_1_, *i*_2__ = 1.
1, b. Assume that *k* < *n* is the largest position such that *R*_1*a*_(*k*) or *R*_1*b*_(*k*) is violated and the condition *R*_2_(*k*) is not satisfied. Then, there is an *i*_1_ ∈ *n*, *i*_1_ ≠ *k*, with *r*_*i*_1_, *k*_ = 0 and *r*_*i*_1_, *n* + 1_ = 1, or there is an *k* < *i*_2_ ≤ *n* such that *r*_*k*,*i*_2__ = 1 and *r*_*n* + 1, *i*_2__ = 0, and we have an *k* < *i*_3_ ≤ *n* with *r*_*i*_3_, *k*_ = 1 and *r*_*n* + 1, *i*_3__ = 1. First, consider the case of *i*_1_ and *i*_3_. If *i*_1_ = *i*_3_, 0 = *r*_*i*_1_, *k*_ = *r*_*i*_3_, *k*_ = 1. Let *i*_1_ ≠ *i*_3_. Since *k* is the largest such critical position, *i*_3_ > *k*, and *r*_*n* + 1, *i*_3__ = 1, the condition *R*_1*a*_(*i*_3_) is fulfilled. Since *i*_1_ ∈ *n*\{*i*_3_} and *r*_*i*_1_, *n* + 1_ = 1, this implies *r*_*i*_1_, *i*_3__ = 1. Since *r*_*n*_ on *n* is transitive and *r*_*i*_3_, *k*_ = 1, we obtain the contradiction 0 = *r*_*i*_1_, *k*_ = 1. Second, consider the case of *i*_2_ and *i*_3_. If *i*_2_ = *i*_3_, 0 = *r*_*n* + 1, *i*_2__ = *r*_*n* + 1, *i*_3__ = 1. Let *i*_2_ ≠ *i*_3_. Since *r*_*n*_ is transitive, and *r*_*i*_3_, *k*_ = 1 and *r*_*k*,*i*_2__ = 1, it holds that *r*_*i*_3_, *i*_2__ = 1. If (*k* <)*i*_3_ < *i*_2_, since *r*_*n* + 1,*i*_3__ = 1, *R*_1*b*_(*i*_3_) holds true. Because *r*_*n* + 1,*i*_2__ = 0, we have *r*_*i*_3_, *i*_2__ = 0. If *i*_2_ < *i*_3_, since *r*_*n* + 1,*i*_2__ = 0, *R*_2_(*i*_2_) is satisfied. Therefore, *r*_*i*_3_, *i*_2__ = 0, because *r*_*n* + 1,*i*_3__ = 1. In both cases, this is in contradiction to *r*_*i*_3_, *i*_2__ = 1.
2, $T\subseteq r_{n}^{⋄}$ Obviously, *r*_*n* + 1_(*x*) is reflexive, and *r*_*n* + 1_(*x*)⋂*n* × *n* = *r*_*n*_. We show that *r*_*n* + 1_(*x*) on *n* + 1 is transitive. We have to distinguish three cases, with *x, y* ∈ *n*, *x* ≠ *y*: (a) *r*_*n* + 1, *x*_ = 1 and *r*_*x, y*_ = 1 implies *r*_*n* + 1, *y*_ = 1, (b) *r*_*x, n* + 1_ = 1 and *r*_*n* + 1, *y*_ = 1 implies *r*_*x, y*_ = 1, and (c) *r*_*x, y*_ = 1 and *r*_*y, n* + 1_ = 1 implies *r*_*x, n* + 1_ = 1.
  Re (a): Let *x* < *y*. Since *r*_*n* + 1, *x*_ = 1 is an admissible value set in the (*n* + 1)th row, *R*_1*b*_(*x*) is true. Because *r*_*x, y*_ = 1, it follows *r*_*n* + 1, *y*_ = 1. Let *y* < *x*. Assume that *r*_*n* + 1, *y*_ = 0. This leads to a contradiction. The condition *R*_2_(*y*) would hold true. Since *r*_*x, y*_ = 1, this would imply *r*_*n* + 1, *x*_ = 0. According to the first part of the proposition, thus *r*_*n* + 1, *y*_ = 1.
  Re (b): The filling *r*_*n* + 1, *y*_ = 1 is admissible and *R*_1*a*_(*y*) is fulfilled. Thus, *r*_*x, n* + 1_ = 1 implies *r*_*x, y*_ = 1.
  Re (c): Let *x* < *y*. The filling *r*_*y, n* + 1_ = 1 in the (*n* + 1)th column is admissible and *C*_1_(*y*) is satisfied. Then, *r*_*x, y*_ = 1 implies *r*_*x, n* + 1_ = 1. Let *y* < *x*. Assume that the *x*th position filled in the (*n* + 1)th column is *r*_*x, n* + 1_ = 0. The condition *C*_2_(*x*) would be fulfilled, and *r*_*x, y*_ = 1 would imply *r*_*y, n* + 1_ = 0. This contradicts the assumption *r*_*y, n* + 1_ = 1. The first part of the proposition yields *r*_*x, n* + 1_ = 1.
2, $r_{n}^{⋄}\subseteq T$. Let $r_{n+1}\in r_{n}^{◇}$. That is, *r*_*n* + 1_ is a quasi-order on {1, …, *n* + 1}, and *r*_*n* + 1_⋂*n* × *n* = *r*_*n*_. Let *x* = (*r*_1, *n* + 1_, …, *r*_*n, n* + 1_, *r*_*n* + 1, *n*_, …, *r*_*n* + 1, 1_), with *r*_*n* + 1_(*x*) = *r*_*n* + 1_, be the relevant entries of *r*_*n* + 1_ the construction needs to retrieve. We show that (a) $\left(r_{1,n+1},r_{2,n+1},\ldots ,r_{n,n+1}\right)\in S_{1}\left(r_{n}\right)$ and (b) $\left(r_{n+1,n},r_{n+1,n-1},\ldots ,r_{n+1,1}\right)\in S_{2}\left(\left(r_{1,n+1},r_{2,n+1},\ldots ,r_{n,n+1}\right),r_{n}\right)$.
  Re (a): Assume that there exists an *k* = 2, …, *n* such that the value *r*_*k, n* + 1_ is not admissible for the *k*th entry of the column *n* + 1, presupposing the given specifications *r*_1, *n* + 1_,…,*r*_*k, n* + 1_ up to this critical position. If *r*_*k, n* + 1_ = 1, *C*_1_(*k*) must be violated. Then, there is an *i* < *k* such that *r*_*i, k*_ = 1 and *r*_*i, n* + 1_ = 0. This contradicts the assumption that *r*_*n* + 1_ is transitive on {1, …, *n* + 1}. If *r*_*k, n* + 1_ = 0, *C*_2_(*k*) is violated. Then, for an *i* < *k*, *r*_*k, i*_ = 1 and *r*_*i, n* + 1_ = 1. However, *r*_*k, n* + 1_ = 0. Thus, $c_{1}\;:=\left(r_{1,n+1},r_{2,n+1},\ldots ,r_{n,n+1}\right)\in S_{1}\left(r_{n}\right)$.
  Re (b): Assume that there exists an *k* = 1, …, *n* such that the value *r*_*n* + 1, *k*_ is not admissible for that entry of the row *n* + 1, conditional on the given values *c*_1_, *r*_*n* + 1, *n*_, *r*_*n* + 1, *n* − 1_, …, *r*_*n* + 1, *k*_. If *r*_*n* + 1, *k*_ = 1, *R*_1*a*_(*k*) or *R*_1*b*_(*k*) must be violated. If *R*_1*a*_(*k*) is not satisfied, there is an *i* ≠ *k* such that *r*_*i, n* + 1_ = 1. However, *r*_*i, k*_ = 0. If *R*_1*b*_(*k*) is not satisfied, there exists an *i* > *k* with *r*_*k, i*_ = 1. However, *r*_*n* + 1, *i*_ = 0. If *r*_*n* + 1, *k*_ = 0, *R*_2_(*k*) must be violated. Then, there is an *i* > *k* such that *r*_*n* + 1, *i*_ = 1 and *r*_*i, k*_ = 1. However, *r*_*n* + 1, *k*_ = 0. Thus, $c_{2}\;:=\left(r_{n+1,n},r_{n+1,n-1},\ldots ,r_{n+1,1}\right)\in S_{2}\left(c_{1},r_{n}\right)$.      □

The overall deterministic construction procedure starts with the set of all quasi-orders $Q_{l}$ on a sufficiently small number of items *l*. Based on the top-down–right-left inductive discrete extension method, every $r_{l}\in Q_{l}$ is extended by one more item. This yields the parent families $r_{l}^{◇}$. Thus, $Q_{l+1}$ is constructed. This process is repeated with $Q_{l+1}$ to generate $Q_{l+2}$, and so forth, until a targeted set $Q_{n}$ of all quasi-orders for some *n* > *l* has been achieved.

## 4. Randomization of the discrete doubly inductive construction procedure

This section introduces a probabilistic modification of the deterministic construction that will be used for the representative sampling of quasi-orders. The general aim is to randomize based on the discrete uniform distribution the construction procedure shown in Figure 1 to transform it into a Laplace random experiment. Another view on the proposed sampling method is to combine the deterministic construction with the uniform extension approach described in Section 2.3. That is, the top-down–right-left method is deployed to correct the random reflexive extensions of the Schrepp and Ünlü (2015) approach that do not satisfy the transitivity property.

### 4.1. Description of the probabilistic sampling procedure

The proposed doubly inductive procedure consists of two levels: the outer Level 2 and inner Level 1 inductive constructions, which are alternated. It starts with a sufficiently small number of items *l* successively extending lower-dimensional trace quasi-orders by one additional item to eventually yield final quasi-orders on a larger number of items *n* > *l* (see Figure 1). It suffices to randomize Level 1 computations, which is the top-down–right-left inductive discrete extension method.

There is a disadvantage of the randomization procedure. The applied corrections are of a combinatorial or non-probabilistic type and entail sampling biases. However, the bias correction factors can be computed based on the following notion of a *biasing position* (see Proposition 4).

**Definition 3**. *Traversing the entries r*_1, *n* + 1_, …, *r*_*n, n* + 1_, *r*_*n* + 1, *n*_, …, *r*_*n* + 1, 1_ *to be filled in the successive order given according to the procedure below, a position of this sequence is called* biasing *if one*, and only one*, of the values* 0 *or* 1 *is admissible for this position*.

**Randomized Level 1 procedure:**

Presuppose a given Level 2 trace quasi-order $r_{n}\in Q_{n}$. To randomly extend it to *n* + 1, *r*_*n* + 1_, we pursue the following strategy (cf. Section 3.1).

1. Randomly fill *r*_1, *n* + 1_ ~ *Bernoulli*(1/2). No checks are necessary. Both of the simulated values 1 and 0 are admissible for this position according to *C*_1_(*k* = 1) and *C*_2_(1), respectively. (Because the position *r*_1, *n* + 1_ can always be set to any of the two values 0 and 1 without violating the transitivity conditions, the first entry always represents a non-biasing position.)
2. Randomly fill *r*_2, *n* + 1_ ~ *Bernoulli*(1/2). The conditions *C*_1_(*k* = 2) and *C*_2_(2) are tested. If the simulated value is admissible, we keep it and proceed to fill the next position. (In this case, the second position may or may not be a biasing position. This depends on whether the *complementary value*, 1 − *r*_2, *n* + 1_, is inadmissible or admissible for this entry, respectively.) If the simulated value is not admissible, 1 − *r*_2, *n* + 1_ is assigned, which necessarily must be admissible according to Proposition 2. (Obviously, the second entry is a biasing position in this case.)
  This process is repeated until the last entry *r*_*n, n* + 1_ ~ *Bernoulli*(1/2) of column *n* + 1 is randomly filled, the conditions *C*_1_(*k* = *n*) and *C*_2_(*n*) are checked, and an admissible value is assigned to this position. Overall, this yields a random vector *c* of admissible values in $S_{1}\left(r_{n}\right)$ fully specifying column *n* + 1.
3. The sampling procedure continues to randomly fill the positions in the (*n* + 1)th row based on the conditions *R*_1*a*_, *R*_1*b*_, and *R*_2_. First, *r*_*n* + 1, *n*_ ~ *Bernoulli*(1/2) needs to be checked for admissibility only if it equals 1. If the sampled value is admissible, we keep that value and continue. Otherwise, if we sampled 1 and *R*_1*a*_(*n*) is not satisfied, the complementary value 0 is admissible (Proposition 2) and assigned to this position.
4. Then, *r*_*n* + 1, *n* − 1_ ~ *Bernoulli*(1/2) is randomly filled. The conditions *R*_1*a*_(*k* = *n* − 1) and *R*_1*b*_(*n* − 1) or *R*_2_(*n* − 1) are examined, and analogously, an admissible value is assigned to this position. This process is repeated until the last entry of row *n* + 1 is filled, *r*_*n* + 1, 1_ ~ *Bernoulli*(1/2). Based on the conditions *R*_1*a*_(*k* = 1) and *R*_1*b*_(1) or *R*_2_(1), the admissibility of the simulated value is checked and, if necessary, replaced by the complementary value.
  This fully specifies the (*n* + 1)th row with a random vector of admissible values in S_2_(*c*, *r_n_*). Thus, the whole relational matrix for *r*_*n* + 1_ has been randomly constructed, which must be a quasi-order on *n* + 1 according to Proposition 2.

An example may be helpful. Consider the quasi-order given by

$$r_{3}=\left[\begin{array}{ccc} 1 & 1 & 1\\ 0 & 1 & 1\\ 0 & 0 & 1 \end{array}\right],$$

which is the relational matrix of a chain structure. Applying the above probabilistic procedure, this quasi-order may be randomly extended by one more item as follows. The first entry is filled with any of the possible Bernoulli realizations, say *r*_1, 4_ = 1,

$$\left[\begin{array}{cccc} 1 & 1 & 1 & \begin{array}{c} 1 \end{array}\\ 0 & 1 & 1 & \\ 0 & 0 & 1 & \\ & & & 1 \end{array}\right].$$

Both 0 and 1 are then admissible values for the entry *r*_2, 4_. The procedure selects one of the two values uniformly at random, say *r*_2, 4_ = 0,

$$\left[\begin{array}{cccc} 1 & 1 & 1 & 1\\ 0 & 1 & 1 & \begin{array}{c} 0 \end{array}\\ 0 & 0 & 1 & \\ & & & 1 \end{array}\right].$$

Given the previous values, we see that for *r*_3, 4_ the only admissible value is 0. This value is assigned,

$$\left[\begin{array}{cccc} 1 & 1 & 1 & 1\\ 0 & 1 & 1 & 0\\ 0 & 0 & 1 & \begin{array}{c} 0 \end{array}\\ & & & 1 \end{array}\right].$$

With this vector of admissible values fully specifying the fourth column, both the values 0 and 1 are admissible for the entry *r*_4, 3_. A Bernoulli realization is taken to fill this entry, say *r*_4, 3_ = 1,

$$\left[\begin{array}{cccc} 1 & 1 & 1 & 1\\ 0 & 1 & 1 & 0\\ 0 & 0 & 1 & 0\\ & & \begin{array}{c} 1 \end{array} & 1 \end{array}\right].$$

For the entry *r*_4, 2_, both the values 0 and 1 are admissible. A value is assigned uniformly at random, say *r*_4, 2_ = 0,

$$\left[\begin{array}{cccc} 1 & 1 & 1 & 1\\ 0 & 1 & 1 & 0\\ 0 & 0 & 1 & 0\\ & \begin{array}{c} 0 \end{array} & 1 & 1 \end{array}\right].$$

Then, for *r*_4, 1_, the only admissible value is 0. This yields

$$r_{4}=\left[\begin{array}{cccc} 1 & 1 & 1 & 1\\ 0 & 1 & 1 & 0\\ 0 & 0 & 1 & 0\\ \begin{array}{c} 0 \end{array} & 0 & 1 & 1 \end{array}\right].$$

In this example, there are two biasing positions. For each of the entries *r*_3, 4_ and *r*_4, 1_, the only admissible value is 0.^4^

The overall probabilistic sampling procedure is a randomized counterpart of the discrete construction procedure shown in Figure 1. It starts with a representative collection of quasi-orders Q(*l*) on a sufficiently small number of items *l*. Applying the randomized top-down–right-left inductive extension, every *r*_*l*_ ∈ Q(*l*) is extended by one more item. Thus, a sample Q(*l* + 1) of random quasi-orders *r*_*l* + 1_ is generated. This process is repeated with *Q*(*l* + 1) to create some Q(*l* + 2), and so forth, until a targeted sample Q(*n*) of random quasi-orders for some *n* > *l* has been achieved.

This sampling procedure can be viewed as a correction technique for the uniform extension approach by Schrepp and Ünlü (2015). This procedure can be used to correct the random extensions that violate the transitivity property. Subsequently, we follow the line of reasoning in the proof of the part “2, $r_{n}^{◇}\subseteq T$” of Proposition 2. In the next section, this result will be used to determine the correction factors needed to balance the sampling biases induced by the combinatorial corrections.

**Correcting random reflexive extensions to satisfy transitivity**, C**:**

Assume that $r_{n+1}^{\prime }$ is a random reflexive, but not necessarily transitive, extension on *n* + 1 of a quasi-order *r*_*n*_ on *n* (in the sense of the definition given in Section 2.3).

Let *r*_1, *n* + 1_, …, *r*_*n, n* + 1_, and *r*_*n* + 1, *n*_, …, *r*_*n* + 1, 1_ be the relevant entries of $r_{n+1}^{\prime }$ that we want to correct if necessary. *In this order*, we successively apply the admissibility tests. Entry for entry, the transitivity conditions *C*_1_, *C*_2_, *R*_1*a*_, *R*_1*b*_, and *R*_2_ are verified. If a value in this sequence is not admissible for the corresponding position (necessarily a biasing position), we replace it with the complementary value. This assigned new value must be admissible (Proposition 2). However, if a value in this sequence is admissible for the corresponding position, we leave it intact. (Such a position may or may not be biasing. If the complementary value is also admissible, this position is non-biasing. Otherwise, it is a biasing position.)

The resulting *corrected* matrix $C\left(r_{n+1}^{\prime }\right)$ is the adjacency matrix of a quasi-order on *n* + 1, unlike $r_{n+1}^{\prime }$ obtained in the original approach by Schrepp and Ünlü (2015). It extends the quasi-order *r*_*n*_ as the trace on *n*.

If we replace one or both of the biasing positions *r*_3, 4_ = 0 or *r*_4, 1_ = 0 of the quasi-order *r*_4_ constructed in the preceding example with the complementary value 1 (in red), the resulting extensions (of *r*_3_)

$$r_{4}^{\prime }:\left[\begin{array}{cccc} 1 & 1 & 1 & 1\\ 0 & 1 & 1 & 0\\ 0 & 0 & 1 & 1\\ 0 & 0 & 1 & 1 \end{array}\right],\left[\begin{array}{cccc} 1 & 1 & 1 & 1\\ 0 & 1 & 1 & 0\\ 0 & 0 & 1 & 0\\ 1 & 0 & 1 & 1 \end{array}\right],\left[\begin{array}{cccc} 1 & 1 & 1 & 1\\ 0 & 1 & 1 & 0\\ 0 & 0 & 1 & 1\\ 1 & 0 & 1 & 1 \end{array}\right]$$

are reflexive but not transitive. Such a matrix may be obtained in the inductive uniform extension approach. We can apply the procedure C to correct for transitivity. For any of these matrices $r_{4}^{\prime }$, it holds that $C\left(r_{4}^{\prime }\right)=r_{4}$.

### 4.2. Induced sampling biases and bias correction factors

This sampling procedure has the advantage that it can generate, very quickly and efficiently, samples of random quasi-orders on very large item sets. The disadvantage is that the combinatorial corrections entail sampling biases in the random process of quasi-order generation. However, as we will discuss next, the induced biases can be corrected.

Why are *sampling biases* induced in this procedure? That is, why can two quasi-orders *r*_*n* + 1_ and *s*_*n* + 1_ with corresponding trace quasi-orders *r*_*n*_ and *s*_*n*_, or similarly, $r_{n+1}^{\left(1\right)}$ and $r_{n+1}^{\left(2\right)}$ with the same trace quasi-order *r*_*n*_, be drawn with different probabilities? The sampling procedure is equivalent to uniformly creating random reflexive extensions in a first probabilistic step, and these extensions are corrected for transitivity using the strategy described in the previous section in a second deterministic step. For an item number *n*, all random reflexive extensions have the same probability 2^−2*n*^ of being drawn. Thus, the probabilities for sampling *r*_*n* + 1_ and *s*_*n* + 1_, or $r_{n+1}^{\left(1\right)}$ and $r_{n+1}^{\left(2\right)}$, are proportional to the numbers of random reflexive extensions that yield the corresponding quasi-orders under reference when corrected according to the procedure. (The same proportionality factor is 2^−2*n*^.) Those sets generally do differ in their cardinalities. However, we can determine their sizes and use this information to adjust for an equal, or approximately equal, sampling probability (Proposition 4).

We require some notation. Let $R_{n+1}$ denote the set of all reflexive relations on *n* + 1. For a trace quasi-order $r_{n}\in Q_{n}$, let $r_{n}^{⊳}:=\left\{r_{n+1}^{\prime }\in R_{n+1}:r_{n+1}^{\prime }⋂\underline{n}\times \underline{n}=r_{n}\right\}$ be the set of all possible random reflexive extensions of *r*_*n*_. The correction of random reflexive extensions described in Section 4.1 can be viewed as the operator $C:r_{n}^{⊳}\to Q_{n+1},r_{n+1}^{\prime }\mapsto C\left(r_{n+1}^{\prime }\right)$. For a sampled quasi-order $r_{n+1}\in r_{n}^{◇}$, let $r_{n}^{⊳}\left[r_{n+1}\right]:=\left\{r_{n+1}^{\prime }\in r_{n}^{⊳}:C\left(r_{n+1}^{\prime }\right)=r_{n+1}\right\}$ denote the set of all random reflexive extensions of the underlying trace quasi-order *r*_*n*_ that yield the quasi-order *r*_*n* + 1_ when corrected according to the correction procedure C.

**Proposition 4**. *Let r*_*n* + 1_ *be a quasi-order randomly generated from a trace quasi-order r*_*n*_ *according to the sampling procedure. It holds that:*

1. *The probability for sampling r*_*n* + 1_ *is*
  $$P\left(r_{n+1}\right)=|r_{n}^{⊳}\left[r_{n+1}\right]|/2^{2n},$$
  *where* $\left|r_{n}^{⊳}\left[r_{n+1}\right]\right|$ *is the number of random reflexive extensions of r*_*n*_ *that, when being corrected using the procedure* C*, yield r*_*n* + 1_.
2. *The size* $\left|r_{n}^{⊳}\left[r_{n+1}\right]\right|$ *can be computed based on the inductive character of the correction. We have*
  $$|r_{n}^{⊳}\left[r_{n+1}\right]|=2^{B\left(r_{n+1}\right)},$$
  *where* 0 ≤ *B*(*r*_*n* + 1_) ≤ 2*n* − 1 *is the number of the biasing positions (Definition 3) among the* 2*n* − 1 *entries r*_2, *n* + 1_, …, *r*_*n, n* + 1_ *and r*_*n* + 1, *n*_, …, *r*_*n* + 1, 1_ *of r*_*n* + 1_ *that have been filled*.

*Proof*. 1. The problem can be framed in complete mathematical form based on probability theory. Let $\left(\Omega^{\prime }:=r_{n}^{⊳},A^{\prime }:=2^{r_{n}^{⊳}},P^{\prime }\right)$ be the Laplace probability space, where Ω′ is the sample or outcome space of random reflexive extensions (of *r*_*n*_), and $A^{\prime }$ is the σ-algebra (power-set) of measurable events or subsets of random reflexive extensions. Since each elementary event or random reflexive extension occurs with the same probability $2^{-2n}=1/|r_{n}^{⊳}|$, this $P^{\prime }:A^{\prime }\to \left[0,1\right]$ is the Laplace probability measure that assigns to each subset of random reflexive extensions, $A\in A^{\prime }$, the probability $P^{\prime }\left(A\right)=|A|/|r_{n}^{⊳}|$. Let $\left(\Omega :=Q_{n+1},A:=2^{Q_{n+1}}\right)$ be the measurable space representing the quasi-orders on *n* + 1. Under these prerequisites, the correction operator $C:r_{n}^{⊳}\to Q_{n+1}$ is a *random variable*, that is, a measurable function mapping the Laplace probability space $\left(\Omega^{\prime },A^{\prime },P^{\prime }\right)$ to the measurable space (Ω,A). Thus, according to probability theory,
  $$\begin{array}{l} P\left(r_{n+1}\right)=P\left(C=r_{n+1}\right):=P\prime \left(\left\{\omega \prime \in \Omega \prime :C\left(\omega \prime \right)=r_{n+1}\right\}\right)\\ \;=P\prime \left(r_{n}^{⊳}\left[r_{n+1}\right]\right). \end{array}$$
2. A position among the filled admissible values *r*_1, *n* + 1_, …, *r*_*n, n* + 1_, *r*_*n* + 1, *n*_, …, *r*_*n* + 1, 1_ of the quasi-order *r*_*n* + 1_ is a biasing position if and only if the value corresponding to this position cannot be replaced by its complementary value, leaving the (preceding) other values of this vector unchanged, without violating the transitivity conditions according to the procedure C. Let *B*(*r*_*n* + 1_) = 0. That is, all positions are non-biasing. We must have $r_{n}^{⊳}\left[r_{n+1}\right]=\left\{r_{n+1}\right\}$. Since any of the two admissible values for each non-biasing position will not be altered under the correction procedure, $r_{n+1}\in r_{n}^{⊳}\left[r_{n+1}\right]$, and $r_{n+1}^{\prime }\in r_{n}^{⊳}\left[r_{n+1}\right]$ implies $r_{n+1}^{\prime }=C\left(r_{n+1}^{\prime }\right)=r_{n+1}$. Thus, $|r_{n}^{⊳}\left[r_{n+1}\right]|=1=2^{B\left(r_{n+1}\right)}$. Let *B*(*r*_*n* + 1_) ≥ 1 biasing positions be denoted by the ordered sequence of their position indices 1 < *b*_1_ < *b*_2_ < … < *b*_*B*(*r*_*n* + 1_)_ ≤ 2*n* among the entries *r*_1, *n* + 1_, …, *r*_*n, n* + 1_, *r*_*n* + 1, *n*_, …, *r*_*n* + 1, 1_ of the quasi-order *r*_*n* + 1_. For ease of notation, for any $r_{n+1}^{\prime }\in r_{n}^{⊳}\left[r_{n+1}\right]$, we refer to its relevant entries $r_{1,n+1}^{\prime }$, $r_{2,n+1}^{\prime }$, …, $r_{n,n+1}^{\prime }$, $r_{n+1,n}^{\prime }$, $r_{n+1,n-1}^{\prime }$, …, $r_{n+1,1}^{\prime }$ as $v_{1}^{\prime }$, $v_{2}^{\prime }$, …, $v_{n}^{\prime }$, $v_{n+1}^{\prime }$, $v_{n+2}^{\prime }$, …, $v_{2n}^{\prime }$, respectively. We show that the projection
  $$\begin{array}{l} p:r_{n}^{⊳}\left[r_{n+1}\right]\to {\left\{0,1\right\}}^{B\left(r_{n+1}\right)},\\ r_{n+1}^{\prime }\mapsto p\left(r_{n+1}^{\prime }\right)\;:=\;\left(v_{b_{1}}^{\prime },v_{b_{2}}^{\prime },\ldots ,v_{b_{B\left(r_{n+1}\right)}}^{\prime }\right) \end{array}$$
  is bijective, thus proving the statement $|r_{n}^{⊳}\left[r_{n+1}\right]|=2^{B\left(r_{n+1}\right)}$.
  Injectivity: Let $r_{n+1}^{\prime },r_{n+1}^{\prime \prime }\in r_{n}^{⊳}\left[r_{n+1}\right]$, $r_{n+1}^{\prime }\neq r_{n+1}^{\prime \prime }$. There is a position with index *i*_0_ such that $v_{i_{0}}^{\prime }\neq v_{i_{0}}^{\prime \prime }$. Suppose *i*_0_ represents a non-biasing position. Then, $v_{i_{0}}^{\prime }$ and $v_{i_{0}}^{\prime \prime }$ are admissible values for this position in matrices $r_{n+1}^{\prime }$ and $r_{n+1}^{\prime \prime }$, respectively. Since the admissible values of a reflexive extension are not altered when corrected, this implies $C\left(r_{n+1}^{\prime }\right)\neq C\left(r_{n+1}^{\prime \prime }\right)$, yielding the contradiction *r*_*n* + 1_ ≠ *r*_*n* + 1_. Thus, *i*_0_ ∈ {*b*_1_, …, *b*_*B*(*r*_*n* + 1_)_}, and $p\left(r_{n+1}^{\prime }\right)\neq p\left(r_{n+1}^{\prime \prime }\right)$.
  Surjectivity: Let $v=\left(v_{b_{1}}^{\prime },v_{b_{2}}^{\prime },\ldots ,v_{b_{B\left(r_{n+1}\right)}}^{\prime }\right)\in {\left\{0,1\right\}}^{B\left(r_{n+1}\right)}$. Replace the entries of the relational matrix *r*_*n* + 1_ at the positions given by the indices *b*_1_ < *b*_2_ < … < *b*_*B*(*r*_*n* + 1_)_ with the values $v_{b_{1}}^{\prime },v_{b_{2}}^{\prime },\ldots ,v_{b_{B\left(r_{n+1}\right)}}^{\prime }$, respectively. For this resulting matrix $r_{n+1}^{\prime }$, we have $r_{n+1}^{\prime }\in r_{n}^{⊳}\left[r_{n+1}\right]$, and $p\left(r_{n+1}^{\prime }\right)=v$.      □

We continue with the previous example. Consider the two quasi-orders

$$r_{4}^{\left(1\right)}=\left[\begin{array}{cccc} 1 & 1 & 1 & 1\\ 0 & 1 & 1 & 0\\ 0 & 0 & 1 & 0\\ 0 & 0 & 1 & 1 \end{array}\right]$$

and

$$r_{4}^{\left(2\right)}=\left[\begin{array}{cccc} 1 & 1 & 1 & 1\\ 0 & 1 & 1 & 0\\ 0 & 0 & 1 & 0\\ 0 & 1 & 1 & 1 \end{array}\right]$$

with the same trace quasi-order

$$r_{3}=\left[\begin{array}{ccc} 1 & 1 & 1\\ 0 & 1 & 1\\ 0 & 0 & 1 \end{array}\right].$$

It holds that $B\left(r_{4}^{\left(1\right)}\right)=2$ and $B\left(r_{4}^{\left(2\right)}\right)=1$; the biasing positions are shown in bold. For their sets of all random reflexive extensions of the underlying trace quasi-order *r*_3_, which yield these quasi-orders when corrected according to the procedure C, we have (inadmissible values highlighted in red)

$$\begin{array}{l} r_{3}^{⊳}\left[r_{4}^{\left(1\right)}\right]=\left\{\left[\begin{array}{cccc} 1 & 1 & 1 & 1\\ 0 & 1 & 1 & 0\\ 0 & 0 & 1 & 0\\ 0 & 0 & 1 & 1 \end{array}\right],\left[\begin{array}{cccc} 1 & 1 & 1 & 1\\ 0 & 1 & 1 & 0\\ 0 & 0 & 1 & 1\\ 0 & 0 & 1 & 1 \end{array}\right],\left[\begin{array}{cccc} 1 & 1 & 1 & 1\\ 0 & 1 & 1 & 0\\ 0 & 0 & 1 & 0\\ 1 & 0 & 1 & 1 \end{array}\right],\left[\begin{array}{cccc} 1 & 1 & 1 & 1\\ 0 & 1 & 1 & 0\\ 0 & 0 & 1 & 1\\ 1 & 0 & 1 & 1 \end{array}\right]\right\} \end{array}$$

and

$$r_{3}^{⊳}\left[r_{4}^{\left(2\right)}\right]=\left\{\left[\begin{array}{cccc} 1 & 1 & 1 & 1\\ 0 & 1 & 1 & 0\\ 0 & 0 & 1 & 0\\ 0 & 1 & 1 & 1 \end{array}\right],\left[\begin{array}{cccc} 1 & 1 & 1 & 1\\ 0 & 1 & 1 & 0\\ 0 & 0 & 1 & 1\\ 0 & 1 & 1 & 1 \end{array}\right]\right\}.$$

These sets do differ in their cardinalities, which are equal to “2 to the power their numbers of the biasing positions.” In particular, the sampling probabilities are $P\left(r_{4}^{\left(1\right)}\right)=1/16$ and $P\left(r_{4}^{\left(2\right)}\right)=1/32$.

The cardinalities determined in Proposition 4 are essential. They can be used as the penalizing weights to adjust for representative, or close to representative, quasi-order sampling. In short, let *r*_*n*+1_ and *s*_*n*+1_ be two quasi-orders generated according to the sampling procedure from their trace quasi-orders *r*_*n*_ and *s*_*n*_. The *bias correction factors* $w_{r_{n+1}}=2^{-B\left(r_{n+1}\right)}$ and $w_{s_{n+1}}=2^{-B\left(s_{n+1}\right)}$ can be used in *post*-construction sampling to equalize the corresponding probabilities. That is, $P\left(r_{n+1}\right)\cdot 2^{-B\left(r_{n+1}\right)}=2^{-2n}$ and $P\left(s_{n+1}\right)\cdot 2^{-B\left(s_{n+1}\right)}=2^{-2n}$. Details are discussed in the following section.

## 5. Procedural variants for bias correction

Three algorithms are introduced to combine the randomized doubly inductive construction with the precise bias correction. The absolute rejection method is the exact approach. However, it is computationally the most intensive. The simple and stratified resampling methods are the recommended procedures. They are computationally viable and efficient, and they provide close to representative random quasi-orders.

### 5.1. Absolute rejection method

The following steps define the *absolute rejection method* (ARM); see Proposition 5. In each inductive step, from *k* to *k* + 1 items, three random experiments are concatenated.

**Random experiment *RE_1_***. The random quasi-orders $r_{k}\in Q_{k}$ are drawn such that they are equally probable.

**Random experiment *RE_2_***. The randomized doubly inductive procedure is applied to construct from the drawn quasi-orders $r_{k}\in Q_{k}$ (random experiment *RE*_1_) the random extensions *r*_*k*+1_ in $Q_{k+1}$ with respective probabilities $P\left(r_{k+1}\right)=2^{B\left(r_{k+1}\right)}/2^{2k}$.

**Random experiment *RE_3_***. After this construction (concatenated random experiment *RE*_2_ ∘ *RE*_1_), a bias-correcting random process $W\sim Bernoulli\left(p=2^{-B\left(r_{k+1}\right)}\right)$ is utilized for *penalizing* the sampled quasi-orders *r*_*k*+1_. If *W* = 1 occurs with probabilities $P\left(W=1\right)=2^{-B\left(r_{k+1}\right)}$, the quasi-orders *r*_*k*+1_ are retained. They are rejected if the outcome *W* = 0 is obtained.

We denote the rejection outcome of the concatenated random experiment *RE*_3_ ∘ (*RE*_2_ ∘ *RE*_1_), that is, of “an inductively constructed quasi-order not being retained,” with symbol $\bar{r}$. Thus, the extended sample spaces for all stages *l* < *i* ≤ *n* of the procedure are given by $\bar{Q_{i}}\;$:$=Q_{i}⋃\left\{\bar{r}\right\}$. (Except for the anchoring or start stage *l*, in which no penalization is required.) For the overall *bias-corrected* sampling procedure, we obtain the representativeness result that is analogous to the main result in Schrepp and Ünlü (2015, p. 4, Proposition).

**Proposition 5**. *Let the bias correction factors be applied to equalize probabilities in repetitions of the randomized Level 1 computations over the Level 2 stages of the randomized doubly inductive procedure from a start stage l (sufficiently small) up to an end stage n* > *l. Then:*

*The final sampling probabilities obtained for the last Level 2 stage n are defined for all of the possible quasi-orders on n items, and all of these probabilities are equal. That is, traversing the proposed hierarchical sampling procedure, we eventually end up with simple random (or uniform) sampling from the quasi-order population* $Q_{n}$.

*Proof*. As anchoring, the procedure starts with some Laplace probability space $\left(\Omega_{l}\;:=Q_{l},A_{l}\;:=2^{Q_{l}},P_{l}\equiv 1/|Q_{l}|\right)$ for a sufficiently small item number *l*. Here, “$P_{l}\equiv 1/|Q_{l}|$” means *P*_*l*_ is defined by $P_{l}\left(r_{l}\right):=1/|Q_{l}|$ for all $r_{l}\in Q_{l}$, and additively extended to $A_{l}$.

In the inductive step, from *l* ≤ *k* < *n* to *k* + 1, assume that we are uniformly sampling from the set of all quasi-orders on *k* items, represented by the Laplace probability space $\left(\Omega_{k}\;:=Q_{k},A_{k}:=2^{Q_{k}},P_{k}\equiv 1/|Q_{k}|\right)$. According to the properties of the deterministic component of the doubly inductive construction procedure (see Section 3.1 and Part 2 of Proposition 2),

$$Q_{k+1}=\underset{r_{k}\in Q_{k}}{\sum }r_{k}^{◇}=\underset{r_{k}\in Q_{k}}{\sum }T\left(r_{k}\right),$$

where “∑ over $r_{k}\in Q_{k}$” stands for the Level 2 construction, and

$$\begin{array}{l} T\left(r_{k}\right)=\left\{r_{k+1}\left(x\right):x=\left(c_{1},c_{2}\right),c_{1}\in S_{1}\left(r_{k}\right),\text{and }c_{2}\in S_{2}\left(c_{1},r_{k}\right)\right\} \end{array}$$

represents the Level 1 construction within a given Level 2 unit $r_{k}\in Q_{k}$. The concatenation *RE*_3_ ∘ (*RE*_2_ ∘ *RE*_1_) can be represented by the probability space $\left(\bar{\Omega_{k\;+\;1}}:=\bar{Q_{k\;+\;1}},\bar{A_{k\;+\;1}}\;:=2^{\bar{Q_{k+1}}},\bar{P_{k\;+\;1}}\right)$. We see from the above deterministic properties that the sample space of all possible outcomes of this concatenated random experiment is the set $\bar{Q_{k+1}}$. According to the formula of total probability, for any $r_{k+1}\in Q_{k+1}$,

$$\begin{array}{ll} \bar{P_{k+1}}\left(r_{k+1}\right) & =\underset{r_{k}^{\prime }\in Q_{k}}{\sum }2^{-B\left(r_{k+1}\right)}P\left(r_{k+1}|r_{k}^{\prime }\right)P\left(r_{k}^{\prime }\right) \end{array}$$

$$\begin{array}{l} \;=2^{-B\left(r_{k+1}\right)}P\left(r_{k+1}|r_{k}\right)P\left(r_{k}\right) \end{array}$$

$$\begin{array}{ll} \;=2^{-B\left(r_{k+1}\right)}\cdot \frac{2^{B\left(r_{k+1}\right)}}{2^{2k}}\cdot \frac{1}{\left|Q_{k}\right|}=\frac{1}{2^{2k}|Q_{k}|},\; & \; \end{array}$$

where $r_{k+1}\in r_{k}^{◇}$ and $P\left(r_{k+1}|r_{k}^{\prime }\right)=0$ for all $r_{k}^{\prime }\in Q_{k}$ with $r_{k}^{\prime }\neq r_{k}$, and

$$\bar{P_{k+1}}\left(\bar{r}\right)=1-\underset{r_{k+1}\in Q_{k+1}}{\sum }\bar{P_{k+1}}\left(r_{k+1}\right)=1-\frac{\left|Q_{k+1}\right|}{2^{2k}|Q_{k}|}.$$

Therefore, for any $r_{k+1}\in Q_{k+1}$, the marginal probability for sampling *r*_*k*+1_ is the same value $2^{-2k}\cdot |Q_{k}{|}^{-1}$. We only focus on and work with the retained inductively constructed quasi-orders. So if we condition on the negation $\neg \bar{r}$, the effective probability for sampling any $r_{k+1}\in Q_{k+1}$ is $\bar{P_{k+1}}\left(r_{k+1}|\neg \bar{r}\right)=\bar{P_{k+1}}\left(r_{k+1}\right)/\left(1-\bar{P_{k+1}}\left(\bar{r}\right)\right)=1/|Q_{k+1}|$. This yields the Laplace probability space $\left(\Omega_{k\;+\;1}\;:=Q_{k\;+\;1},A_{k\;+\;1}\;:=2^{Q_{k+1}},P_{k\;+\;1}\equiv 1/|Q_{k\;+\;1}|\right)$, which represents simple random sampling from the quasi-order population $Q_{k+1}$.      □

### 5.2. Simple and stratified resampling methods

The *simple resampling method* (SIRM) and the *stratified resampling method* (STRM) are approximate, sufficiently precise variants for bias correction. Their usefulness is demonstrated based on simulation studies (Section 6). The theoretical study of the probability theory foundation of the SIRM and STRM and of their interrelationship require more work, which is an interesting direction for future research (cf. Section 7).

#### 5.2.1. SIRM approach

The SIRM is anchored with simple random sampling for a small item number *l*. That is, we start with a Laplace probability space $\left(\Omega_{l}:=Q_{l},A_{l}\;:=2^{Q_{l}},P_{l}\equiv 1/|Q_{l}|\right)$. In each inductive step of the doubly inductive procedure, from *l* ≤ *k* < *n* to *k* + 1 items, the SIRM is the concatenation of the following random experiments.

First, we run the *construction* component. A bias-corrected sample (explained below) of a fixed size *N*, denoted by Q_*N*_(*k*), of approximately representative to representative random quasi-orders on *k* items is presupposed and extended based on the randomized doubly inductive construction procedure. For the anchoring *k* = *l*, Q_*N*_(*l*) is any simple random sample of size *N* drawn with replacement (or without, if possible) from the known quasi-order population $Q_{l}$. That is, the randomized doubly inductive construction procedure is applied to extend each quasi-order *r*_*k*_ of the sample and multiset (possibly with repetitions) Q_*N*_(*k*) to a random quasi-order $r_{k+1}\in Q_{k+1}$ with probability $P\left(r_{k+1}\right)=2^{B\left(r_{k+1}\right)}/2^{2k}$. We collect all of these extensions *r*_*k*+1_ in a *constructed* multiset of size *N*, denoted by $Q_{N}^{\prime }\left(k+1\right)$.

Second, with the *correction* component, the constructed sample $Q_{N}^{\prime }\left(k+1\right)$ is corrected for biases. This is achieved by weighted resampling with replacement. The weight assigned to an element *r*_*k*+1_ of $Q_{N}^{\prime }\left(k+1\right)$ is

$$\frac{2^{-B\left(r_{k+1}\right)}}{\sum_{r_{k+1}^{\prime }\in Q_{N}^{\prime }\left(k+1\right)}2^{-B\left(r_{k+1}^{\prime }\right)}}.$$

These are the probability weights for obtaining the quasi-orders of $Q_{N}^{\prime }\left(k+1\right)$. The resulting resample and multiset of the fixed size *N* is the *bias-corrected* sample obtained for the induction step *k* + 1 of the SIRM. It consists of close to representative random quasi-orders on *k* + 1 items, denoted by Q_*N*_(*k* + 1).

#### 5.2.2. STRM approach

The STRM is anchored with simple random sampling for a feasibly small item number *l*, that is, with some Laplace probability space $\left(\Omega_{l}:=Q_{l},A_{l}:=2^{Q_{l}},P_{l}\equiv 1/|Q_{l}|\right)$. In each inductive step of the doubly inductive procedure, from *l* ≤ *k* < *n* to *k* + 1 items, the STRM is the concatenation of the following random experiments.

The first step of the STRM equals the SIRM. As the *construction* component, a bias-corrected sample Q_*N*_(*k*) of a fixed size *N* of close to representative or representative random quasi-orders on *k* items is extended based on the randomized doubly inductive construction procedure. As the anchoring, Q_*N*_(*l*) is a simple random sample of size *N* drawn with (or without) replacement from the quasi-order population $Q_{l}$. All extensions $r_{k+1}\in Q_{k+1}$ of the quasi-orders *r*_*k*_ ∈ Q_*N*_(*k*) are collected in a *constructed* sample $Q_{N}^{\prime }\left(k+1\right)$ of size *N*.

In their second steps, the STRM and SIRM do differ. The correction component of the STRM is an approach based on *stratification*, whereby the biased constructed multiset $Q_{N}^{\prime }\left(k+1\right)=Q^{\prime }$ is partitioned into specific submultisets or strata. Let

$$B_{Q^{\prime }}:=\left\{b=B\left(r_{k+1}\right):r_{k+1}\in Q_{N}^{\prime }\left(k+1\right)\right\}$$

be the set of the *unique* numbers of the biasing positions implied by the quasi-orders in the sample $Q_{N}^{\prime }\left(k+1\right)$; see Definition 3 and Proposition 4. The family

$$S\;:=\;\left\{S_{b}:b\in B_{Q^{\prime }}\right\}$$

is a partition of the sample $Q_{N}^{\prime }\left(k+1\right)$, where

$$S_{b}\;:=\;\left\{r_{k+1}\in Q_{N}^{\prime }\left(k+1\right):B\left(r_{k+1}\right)=b\right\}$$

is the submultiset of quasi-orders in $Q_{N}^{\prime }\left(k+1\right)$ with the same number *b* of their biasing positions. The partition elements $S_{b}\in S$ for $b\in B_{Q^{\prime }}$ are called *strata*. Thus, the strata are defined *ex post* in the constructed sample, based on the numbers of the biasing positions obtained for the sampled quasi-orders. We denote with |*S*_*b*_| the cardinality of a stratum *S*_*b*_, that is, the total number of elements including repeated membership. Note that |*S*_*b*_| is the absolute frequency of how often the number $b\in B_{Q^{\prime }}$ of biasing positions is observed in the sample $Q_{N}^{\prime }\left(k+1\right)$.

With the *correction* component of the STRM, the constructed sample $Q_{N}^{\prime }\left(k+1\right)$ is corrected for biases. This is achieved by weighted resampling after stratification, followed by simple random sampling within the drawn strata. Both the resampling and sampling occur with replacement. By definition, the strata uniquely correspond to the numbers of biasing positions. Thus, weighting and resampling of the strata $S_{b}\in S$ can be implemented by weighting and resampling the elements of the set $B_{Q^{\prime }}$. The weight assigned to an element *b* of $B_{Q^{\prime }}$ is

$$\frac{|S_{b}|\cdot 2^{-b}}{\sum_{b^{\prime }\in B_{Q^{\prime }}|S_{b^{\prime }}|\cdot 2^{-b^{\prime }}}}.$$

These are the probability weights for obtaining the elements of $B_{Q^{\prime }}$. Let this sample be denoted by *B*_*S*_ (|*B*_*S*_| = *N*).

For any *b* in *B*_*S*_, including repeated membership, consider the uniquely determined stratum *S*_*b*_. All quasi-orders of this “drawn” multiset *S*_*b*_ have the same sampling probability 1/|*S*_*b*_|. From *S*_*b*_, one element is randomly selected. This can be equivalently formulated as follows. Let $B_{S}^{\prime }$ be the underlying set of the unique elements of the multiset *B*_*S*_. That is, only one instance of an element is allowed. For every $b^{*}\in B_{S}^{\prime }$, let the multiplicity or number of occurrences of b^*^ in the multiset *B*_*S*_ be denoted with *m*(b^*^) ≥ 1 ($\underset{b^{*}\in B_{S}^{\prime }}{\sum }m\left(b^{*}\right)=N$). From each stratum $S_{b^{*}}$ for $b^{*}\in B_{S}^{\prime }$, a simple random sample with replacement of size *m*(b^*^) is drawn. All resampled quasi-orders are put together. The resulting multiset of size *N*, of close to representative random quasi-orders on *k* + 1 items, is the *bias-corrected* sample $Q_{N}\left(k+1\right)$ obtained for the induction step *k* + 1 of the STRM.

Section 6 reports the simulation results demonstrating the usefulness of the SIRM and STRM approaches for representative quasi-order sampling.

## 6. Simulation results

We present simulation results for the ARM, SIRM, and STRM used to sample quasi-orders. The representativeness of the quasi-order samples was assessed using as an evaluation criterion the size or cardinality of a quasi-order. In addition, the *per–hundred–quasi-orders* mean computing time in seconds (*s*) required on average for randomly generating 100 quasi-orders is reported. We also computed the Tukey (1977) *five-number summaries* (here, box plot statistics), the lower-whisker extreme, lower-hinge (first quartile), median, upper-hinge (third quartile), and the upper-whisker extreme. Moreover, the mean, scatter plot, histogram, and kernel density estimate of the sample quasi-order sizes are presented. These summary measures are used to describe and visualize their distribution. The whiskers of the box plot extend to the most extreme data points that are no more than 1.5 times the interquartile range, or the length of the box, away from the box edges. Thus, the box plot represents both the summary statistics about center and spread and the distribution of the primary data (Tukey, 1977).

The computations were run in R (The R Core Team, 2016, www.R-project.org) on an Intel Core i7 3.4 GHz processor. Throughout the simulation studies, in all of the cases, the inductive construction processes were anchored by using the population of all four (labeled) quasi-orders on *l* = 2 items.

### 6.1. Assessing representativeness in the complete inventory cases of *n* = 3, 4, 5, and 6 items

First, we evaluate the quality of the representativeness of the sampling techniques for *n* = 3, 4, 5, and 6 items. For these item numbers, the complete population of all quasi-orders can be constructed reasonably quickly. Thus, the created samples can be compared to the true population properties. On a set of *n* = 3, 4, 5, and 6 items, there exist 29, 355, 6942, and 209, 527 (labeled) quasi-orders, respectively (e.g., Brinkmann and McKay, 2002). These populations were known and were used in the following analyses.

**Figure 2:**
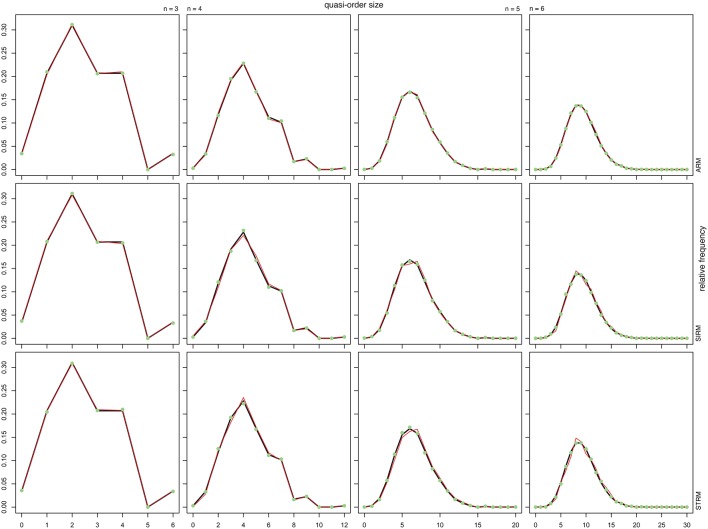
**The relative frequencies of the quasi-order sizes (excluding the reflexive item pairs) computed in the populations of all quasi-orders (solid dark line), which are compared with the means of the relative frequencies of the sizes computed over 100 trials in each of the samples of 100 (solid red line) and 500 (filled green dot) quasi-orders**. From left to right, the first, second, third, and fourth columns stand for *n* = 3, 4, 5, and 6 items, respectively. From top to bottom, the first, second, and third rows represent the ARM, SIRM, and STRM, respectively. In each of these cases, we started the inductive construction anchoring with *l* = 2 items.

Figure 2 shows the sample (solid red line or filled green dot) and population (solid dark line) distributions of the quasi-order sizes (without the reflexive item pairs). The reported sample values are the averages taken over 100 trials or quasi-order samples drawn according to the respective methods. The population values were computed in the given sets of all possible quasi-orders. Two sample sizes of randomly generated quasi-orders were considered. In Figure 2, the solid red line and filled green dot are for quasi-order samples of sizes *N* = 100 and 500, respectively. The columns stand for *n* = 3, 4, 5, and 6 items, and the rows represent the sampling methods ARM, SIRM, and STRM, respectively.

From Figure 2, we see that under any method, the true distributions were estimated very well for all item numbers and with especially higher accuracy as the sample size increased. In contrast to the practicable resampling methods SIRM and STRM, the theoretical rejection method ARM yielded more representative quasi-order samples with smaller sample sizes. However, we will demonstrate in the following section that this result is obtained with substantial extra computation cost when more items are used.

### 6.2. Assessing representativeness in comparison to Schrepp and Ünlü (2015) or up to *n* = 20 items

**Figure 3:**
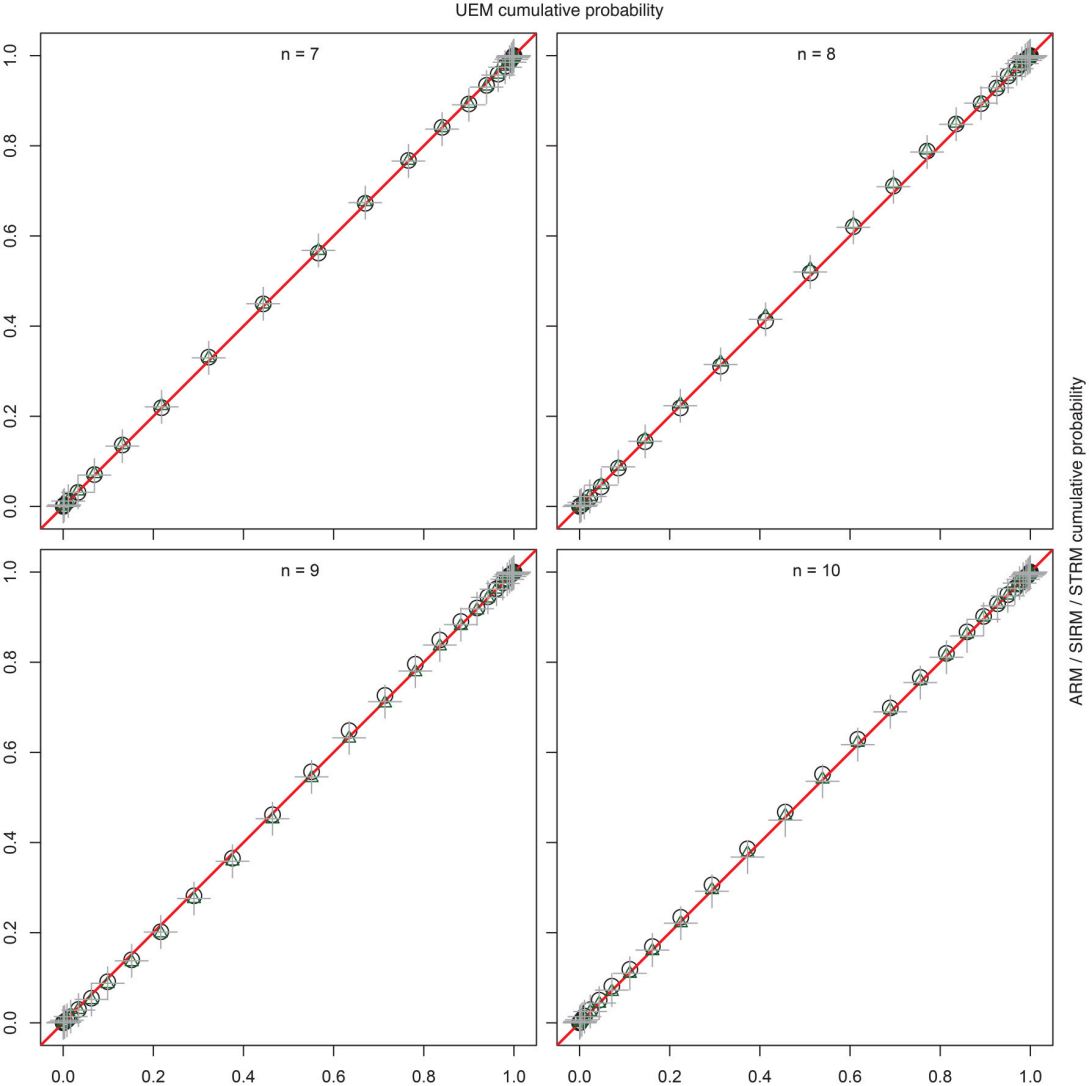
**For item numbers ***n*** = 7, 8, 9, and 10, ***P***-***P*** plots are shown comparing the empirical cumulative distribution functions of the sample quasi-order sizes for the ARM, SIRM, and STRM (***y***-axes) to the cumulative distribution functions for the UEM as the references (***x***-axes)**. All methods were anchored with *l* = 2 items. The empirical cumulative distribution functions were evaluated at the potential knots or sizes 0, 1, …, *n*^2^ − *n* (without the reflexive item pairs). They represent mean cumulative probabilities taken over the samples (ARM, UEM: 10 trials, *N* = 1000; SIRM, STRM: 50 trials, *N* = 10, 000). The plotting symbols used for the ARM, SIRM, and STRM are unfilled black circles, unfilled green triangles, and gray plus-signs, respectively. All points fall on the comparison lines *y* = *x* (in red), which indicates that the four methods yield virtually the “same” and representative size sampling distributions.

For comparison with the UEM by Schrepp and Ünlü (2015), Figure 3 shows the percent-percent (*P*-*P*) plots (e.g., Tukey, 1977). These plots compare the empirical cumulative distribution functions of the sample quasi-order sizes for ARM, SIRM, and STRM, placed on the *y*-axes, with the sample cumulative probabilities for the quasi-order sizes observed under the UEM, as the reference distribution functions placed on the *x*-axes. The straight lines in red, *y* = *x*, are used for comparison. Deviations of the points from the lines indicate differences between the two distributions being plotted against each other. The comparisons were made for the item numbers *n* = 7, 8, 9, and 10. For the computationally intensive methods ARM and UEM, ten trials each with *N* = 1000 quasi-orders were run, compared to the much faster SIRM and STRM procedures, with 50 trials each of *N* = 10, 000 simulated quasi-orders. The cumulative probabilities graphed in Figure 3 are the mean values computed over the trials, where the empirical cumulative distribution functions were evaluated at the potential and unique quasi-order sizes 0, 1, …, *n*^2^ − *n* (i.e., excluding the reflexive item pairs).

From Figure 3, we can see that the points in the *P*-*P* plots all fall on the straight lines (in red). This indicates that the sampling methods ARM, SIRM, STRM, and UEM give virtually the “same” size distributions for quasi-orders being randomly and representatively generated by any of these methods. In particular, we conclude that the fast resampling-based SIRM and STRM methods in the studied simulation conditions yielded representative quasi-order samples by comparison with such theoretically exact, but computationally intensive, procedures as the ARM and UEM.

Under any method, the per–hundred–quasi-orders mean computing time (in *s*) is shown in Table 1. The time required on average was calculated over 100 trials of quasi-order samples of the size *N* = 100. The UEM and ARM methods are computationally intensive. This result is strikingly highlighted by the per–hundred–quasi-orders mean computing time reported for *n* = 13 and 14 items under the UEM in Table 1. These times were computed over five trials each of *N* = 1000 simulated quasi-orders.

**Table 1:** **Per–hundred–quasi-orders mean computing time (in ***s***) calculated over 100 trials of quasi-order samples of size ***N*** = 100 for item numbers ***n*** = 7, …, 20 (SIRM and STRM), ***n*** = 7, …, 12 (UEM), and ***n*** = 7, …, 10 (ARM)**.

***n***	**Computing time (in** ***s*****)**			
	**SIRM**	**STRM**	**UEM**	**ARM**
7	0.044 (0.001)	0.047 (0.001)	0.399 (0.032)	1.751 (0.130)
8	0.061 (0.006)	0.064 (0.005)	1.137 (0.092)	5.715 (0.473)
9	0.080 (0.004)	0.083 (0.003)	3.226 (0.289)	18.636 (1.462)
10	0.102 (0.006)	0.107 (0.005)	9.270 (0.795)	59.088 (5.121)
11	0.127 (0.005)	0.132 (0.004)	26.990 (1.971)	
12	0.156 (0.006)	0.161 (0.005)	80.547 (6.104)	
13	0.188 (0.009)	0.194 (0.009)	235.516 (4.190)	
14	0.224 (0.009)	0.233 (0.009)	724.929 (14.922)	
15	0.264 (0.011)	0.271 (0.008)		
16	0.305 (0.013)	0.316 (0.011)		
17	0.356 (0.015)	0.365 (0.011)		
18	0.407 (0.014)	0.419 (0.012)		
19	0.463 (0.014)	0.477 (0.013)		
20	0.521 (0.016)	0.538 (0.015)		

**Figure 4:**
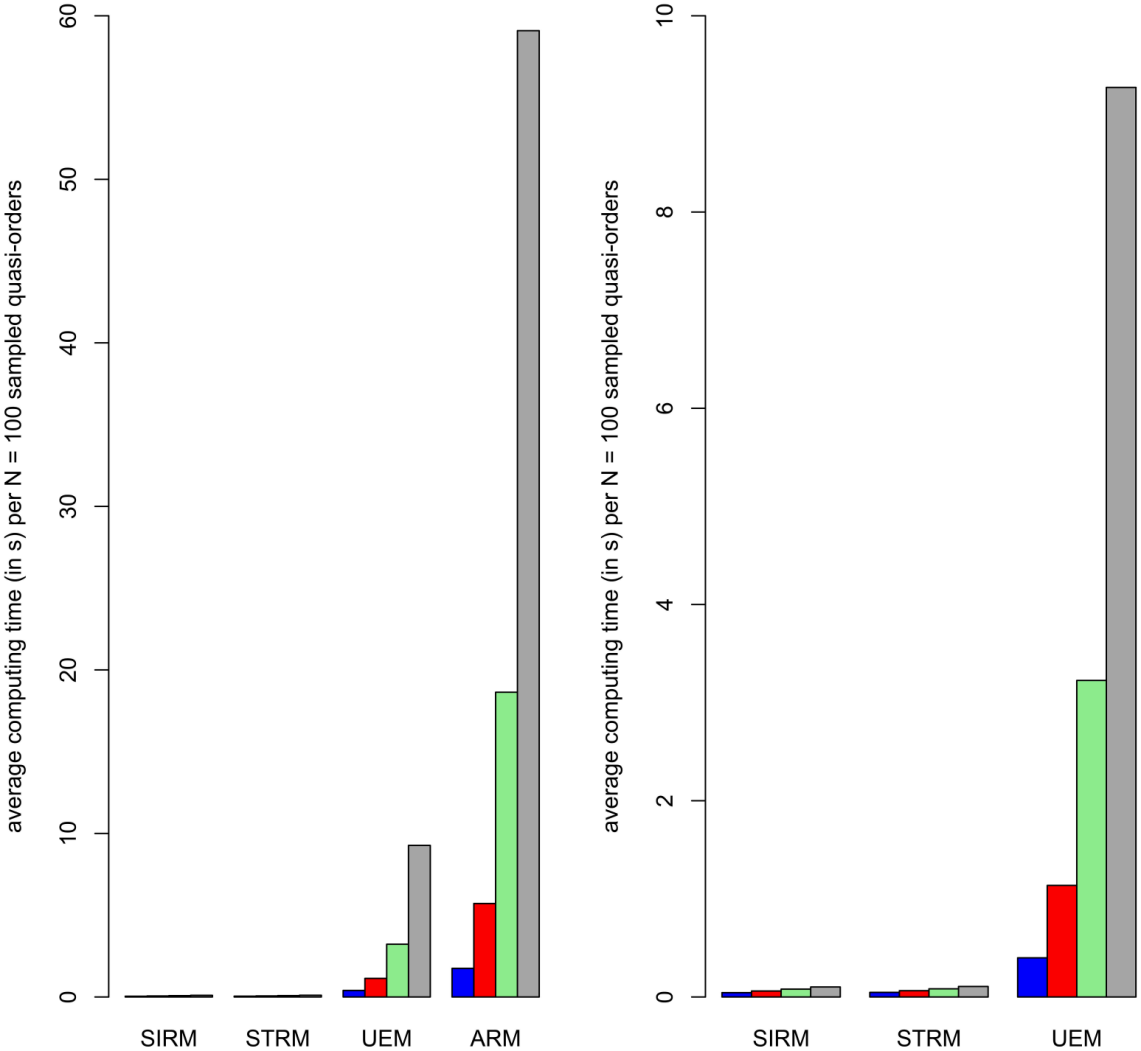
**Bar plot representations of the average computing times (in ***s***) for randomly generating a sample of ***N*** = 100 quasi-orders under any of the four methods (cf. Table 1)**. From left to right, the juxtaposed bars within a method represent the computing times obtained for item numbers *n* = 7, …, 10 in blue, red, green, and gray colors, respectively. The second column plot zooms in on the fast computing times achieved with the SIRM and STRM. It omits plotting the most intensive computing times required by the ARM.

*To illustrate the computational intensity of the UEM (and, thus, of the ARM), the average times required to randomly generate a sample of 100 quasi-orders under the UEM were also run for n = 13 and 14 items. These values were calculated from five quasi-order samples of size N = 1000. Standard deviations are in parentheses. In any of the cases, the inductive construction was anchored with the set of all four quasi-orders on l = 2 items. The average computing times recorded for the joint item numbers are plotted in Figure 4*.

The joint results shown in Table 1 are visualized using bar plot representations in Figure 4. The juxtaposed bars depict the average computing times, in respective order, obtained for the item numbers *n* = 7, …, 10 under the different methods.

As can be seen from Table 1 or Figure 4, the SIRM and STRM methods were very fast. The ARM and UEM required considerably higher computing times. Worst in this regard was the ARM, followed by the UEM. We observed that the ARM first ran into a relatively longer computing time, requiring one-and-a-half hours or more, with the item number *n* = 10. Such a critical threshold for the UEM was attained with *n* = 12 items. In this sense, the UEM may be said to be “Δ*n* = 2 items ahead of” the ARM. Throughout the simulation studies, the SIRM and STRM methods generally gave comparable results. The time savings with the SIRM and STRM are significant. In Section 6.3, we will use these methods to construct exemplary close to representative quasi-order samples for item numbers as high as *n* = 20, 30, 40, and 50. Even more is conceivable. This could not be realized with any of the other approaches.

In Table 2, we catalog the five-number summaries and the means of the sizes of randomly generated quasi-orders, shown for item numbers *n* = 11, …, 20.

**Table 2:** **Averaged five-number summaries (with whiskers as defined) and arithmetic means of the sizes (excluding the reflexive item pairs) of randomly generated quasi-orders for item numbers ***n*** = 11, …, 14 (UEM) and ***n*** = 11, …, 20 (SIRM and STRM)**.

***n***	**SIRM**	**STRM**	**UEM**	**SIRM**	**STRM**	**UEM**
	**Median (first line)/Mean (second line)**			**Lower-whisker (first line)/Upper-whisker (second line)**		
11	27.330 (0.667)	27.490 (0.643)	27.300 (0.483)	13.810 (1.143)	13.770 (1.136)	14.100 (1.287)
	27.580 (0.523)	27.723 (0.519)	27.609 (0.245)	41.600 (1.414)	41.940 (1.324)	41.500 (1.080)
12	31.980 (0.841)	32.220 (0.675)	32.000 (0.816)	17.380 (1.516)	17.360 (1.501)	17.700 (1.418)
	32.288 (0.661)	32.470 (0.587)	32.146 (0.704)	47.250 (1.513)	47.700 (1.605)	46.900 (1.729)
13	37.095 (0.987)	37.550 (0.702)	37.200 (0.447)	21.020 (1.639)	21.290 (1.409)	21.000 (1.581)
	37.383 (0.829)	37.830 (0.608)	37.238 (0.334)	53.970 (1.867)	54.460 (1.702)	54.600 (1.140)
14	42.430 (1.139)	42.980 (0.910)	43.000 (0.707)	25.550 (1.749)	25.320 (1.842)	26.000 (1.225)
	42.812 (0.951)	43.242 (0.832)	43.097 (0.478)	60.410 (2.252)	61.190 (1.813)	60.000 (0.707)
15	48.500 (0.850)	48.600 (0.966)		29.400 (1.776)	28.600 (2.797)	
	48.762 (0.845)	48.861 (0.670)		68.500 (1.509)	68.500 (1.509)	
16	55.000 (1.764)	55.000 (0.667)		34.300 (3.773)	34.500 (2.121)	
	55.187 (1.480)	55.204 (0.563)		76.000 (3.197)	75.900 (2.424)	
17	61.100 (0.994)	60.900 (1.524)		40.600 (2.591)	39.400 (1.955)	
	61.216 (1.125)	61.446 (1.250)		81.900 (3.035)	83.400 (2.011)	
18	67.740 (1.352)	68.420 (0.971)		44.740 (2.601)	44.480 (2.140)	
	68.090 (1.142)	68.587 (0.993)		91.260 (1.998)	92.580 (1.774)	
19	74.760 (1.611)	75.380 (1.159)		51.140 (3.375)	50.560 (2.426)	
	75.135 (1.405)	75.677 (0.932)		99.080 (4.080)	100.640 (2.202)	
20	82.300 (2.697)	82.700 (1.594)		56.820 (3.713)	56.260 (4.388)	
	82.495 (2.427)	82.907 (1.662)		108.380 (4.742)	109.620 (3.463)	
	**Lower-hinge (version of first quartile)**			**Upper-hinge (version of third quartile)**		
11	23.745 (0.796)	23.900 (0.732)	23.900 (0.316)	31.000 (0.569)	31.230 (0.584)	31.100 (0.316)
12	28.250 (0.845)	28.430 (0.795)	28.300 (0.823)	35.950 (0.702)	36.230 (0.679)	35.900 (0.738)
13	33.005 (1.116)	33.420 (0.755)	32.600 (0.548)	41.490 (0.969)	41.955 (0.779)	41.600 (0.548)
14	38.140 (1.206)	38.550 (1.058)	38.600 (0.894)	47.170 (1.104)	47.700 (0.969)	47.400 (0.548)
15	43.700 (1.160)	43.500 (1.269)		53.700 (0.483)	53.600 (0.516)	
16	49.600 (2.066)	49.900 (0.738)		60.300 (1.418)	60.400 (0.843)	
17	55.800 (1.549)	55.600 (1.578)		66.300 (1.567)	66.800 (1.476)	
18	62.080 (1.536)	62.320 (1.285)		73.860 (1.229)	74.560 (1.033)	
19	68.880 (1.837)	69.240 (1.255)		81.040 (1.829)	81.900 (1.093)	
20	75.780 (2.750)	76.020 (2.575)		88.940 (2.831)	89.540 (1.446)	

**Figure 5:**
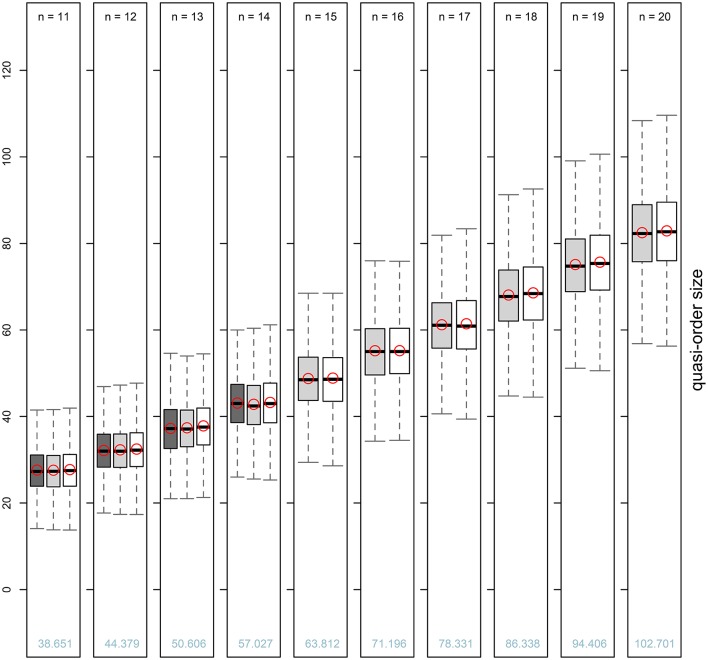
**Box plot representations for the averaged five-number summaries and arithmetic means of the sizes of randomly generated quasi-orders (see Table 2) for item numbers ***n*** = 11, …, 14 under the UEM (dark gray boxes) and ***n*** = 11, …, 20 under the SIRM and STRM (light gray and white boxes, respectively)**. The “within-method” average mean values are plotted as unfilled red circles in their respective boxes. The numerical values of the “across-methods” overall means of the quasi-order sizes (including the reflexive item pairs) averaged over the SIRM and STRM are printed as light blue figures for the different item numbers.

*For the UEM, the average values were calculated using ten (n = 11,12) or five (n = 13, 14) quasi-order samples of size N = 1000. For the SIRM and STRM, we used 100 samples each with N = 10,000 simulated quasi-orders (n = 11, …, 14), ten quasi-order samples of size N = 20, 000 (n = 15, …, 17), and 50 samples of N = 50,000 quasi-orders (n = 18, …, 20). Standard deviations are in parentheses. The inductive construction was always anchored with l = 2 items. The results are plotted in Figure 5*.

The box plot statistics and the means reported in Table 2 are visualized in Figure 5.

From Table 2 or Figure 5, we can see that the fast methods SIRM and STRM yielded quasi-order samples that were close to the theoretically representative samples of the exact UEM method. The SIRM and STRM could be well matched in simulation yielding comparable results. This leads to concurrent and agreeing evidence for the representativeness of the obtained results. Compared to the whisker extremes, the location measures median and mean and the interquartile range as the spread were similar and exhibited less variation across the different methods.

In Section 6.3, the median and mean quasi-order sizes will be extended to larger item numbers. The cataloged location estimates represent useful information and may be referenced as benchmarking figures for the quick and frugal evaluation of the representativeness of candidate sets of quasi-orders. In addition, we will present scatter plots, histograms, and kernel density estimates for the nuanced visualization of the quasi-order samples obtained from the SIRM and STRM procedures.

### 6.3. Resampling-based quasi-order samples on up to *n* = 50 items

In Table 3, we catalog the mean and median quasi-order sizes for *n* = 3, …, 50 items estimated under the SIRM and STRM. For each item number, one sample of *N* = 500, 000 quasi-orders was randomly drawn according to the SIRM and STRM. The true population values known for *n* = 3, …, 6 are highlighted.

**Table 3:** **The mean and median (in parentheses) quasi-order sizes (including the reflexive item pairs) estimated under the SIRM and STRM for item numbers up to ***n*** = 50**.

***n***	**SIRM**	**STRM**	**Average**	***n***	**SIRM**	**STRM**	**Average**
	**Mean (median) quasi-order size**				**Mean (median) quasi-order size**		
3	5.484 (5)	5.481 (5)	5.483 (5)	27	171.626 (171)	172.892 (173)	172.259 (172)
	**5.483** (**5**)	**5.483** (**5**)	**5.483** (**5**)				
4	8.362 (8)	8.354 (8)	8.358 (8)	28	182.682 (182)	184.061 (184)	183.372 (183)
	**8.361** (**8**)	**8.361** (**8**)	**8.361** (**8**)				
5	11.625 (11)	11.597 (11)	11.611 (11)	29	194.923 (195)	195.917 (196)	195.420 (195.500)
	**11.612** (**11**)	**11.612** (**11**)	**11.612** (**11**)				
6	15.226 (15)	15.199 (15)	15.213 (15)	30	206.586 (205)	207.132 (207)	206.859 (206)
	**15.220** (**15**)	**15.220** (**15**)	**15.220** (**15**)				
7	19.203 (19)	19.142 (19)	19.173 (19)	31	218.906 (218)	219.527 (220)	219.217 (219)
8	23.524 (23)	23.437 (23)	23.481 (23)	32	232.688 (232)	231.914 (231)	232.301 (231.500)
9	28.177 (28)	28.120 (28)	28.149 (28)	33	246.191 (246)	245.808 (246)	245.9995 (246)
10	33.209 (33)	33.136 (33)	33.173 (33)	34	258.706 (258)	258.860 (259)	258.783 (258.500)
11	38.522 (38)	38.553 (38)	38.538 (38)	35	272.967 (272)	272.386 (273)	272.677 (272.500)
12	44.259 (44)	44.193 (44)	44.226 (44)	36	286.309 (286)	286.839 (288)	286.574 (287)
13	50.289 (50)	50.287 (50)	50.288 (50)	37	302.229 (302)	302.259 (303)	302.244 (302.500)
14	56.734 (56)	56.454 (56)	56.594 (56)	38	317.764 (320)	317.773 (318)	317.769 (319)
15	63.555 (63)	63.222 (63)	63.389 (63)	39	334.588 (335)	334.834 (336)	334.711 (335.500)
16	70.734 (70)	70.217 (70)	70.476 (70)	40	352.186 (352)	352.304 (353)	352.245 (352.500)
17	77.878 (78)	77.453 (77)	77.666 (77.500)	41	369.235 (368)	369.636 (371)	369.436 (369.500)
18	85.718 (85)	85.415 (85)	85.567 (85)	42	387.988 (388)	387.459 (389)	387.724 (388.500)
19	93.794 (94)	93.395 (93)	93.595 (93.500)	43	405.442 (405)	405.446 (407)	405.444 (406)
20	102.028 (102)	101.954 (102)	101.991 (102)	44	424.055 (423)	423.938 (426)	423.997 (424.500)
21	111.359 (111)	111.011 (111)	111.185 (111)	45	442.233 (442)	441.528 (444)	441.881 (443)
22	119.345 (119)	119.923 (120)	119.634 (119.500)	46	461.318 (461)	461.070 (461)	461.194 (461)
23	128.722 (128)	129.952 (130)	129.337 (129)	47	479.437 (481)	479.874 (480)	479.656 (480.500)
24	138.617 (138)	140.088 (140)	139.353 (139)	48	497.398 (496)	497.605 (498)	497.502 (497)
25	149.458 (149)	150.209 (151)	149.834 (150)	49	515.797 (518)	515.774 (516)	515.786 (517)
26	160.060 (160)	161.311 (162)	160.686 (161)	50	534.039 (537)	534.276 (534)	534.158 (535.500)

**Figure 6:**
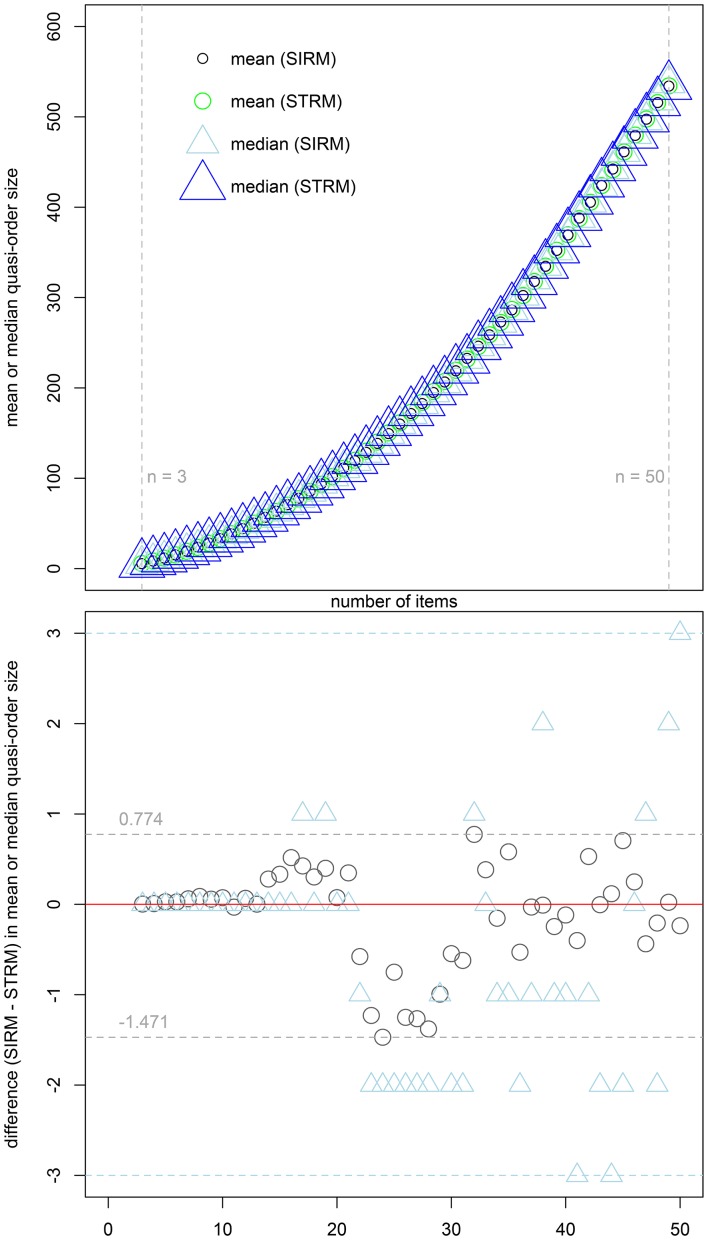
**The first row scatter plot shows the mean quasi-order sizes (unfilled black and green circles for the SIRM and STRM, respectively) and median quasi-order sizes (unfilled light blue and blue triangles for the SIRM and STRM, respectively) as a function of item numbers ***n*** = 3, …, 50 (***x***-axis)**. The reflexive item pairs are included. The values were computed in the SIRM and STRM samples, each having half-a-million quasi-orders (cf. Table 3). The second row scatter plot zooms in to the differences in mean and median quasi-order sizes, here of values obtained under the SIRM minus the corresponding values for the STRM. The differences in mean and median are depicted as unfilled dark gray circles and unfilled light blue triangles, respectively. Their minima and maxima are represented by the horizontal dashed lines of the same colors.

*For any n = 3, …, 50, one sample of N = 500,000 quasi-orders was constructed based on each method. The average values of the mean and median quasi-order sizes taken over both methods can be found in the “Average” columns. The true population values known for n = 3, …,6 are shown in bold. In each case, the inductive construction was anchored with l = 2 items. The results are plotted in Figure 6*.

With larger item numbers, we have seen substantial variability in the location estimates. Greater sample sizes or many repetitions may be necessary to control for such instability effects. These effects may particularly arise when point-estimating the population mean quasi-order sizes. However, the SIRM and STRM sampling techniques could be well matched in the simulation study, thus leading to culminating evidence. From Figure 6, we see that the mean and median values reported in Table 3 were very close or comparable. The resulting graph for the mean quasi-order size, as a function of the item number, seems to be following a quadratic polynomial function. Future research into these issues is needed (cf. Section 7).

**Figure 7:**
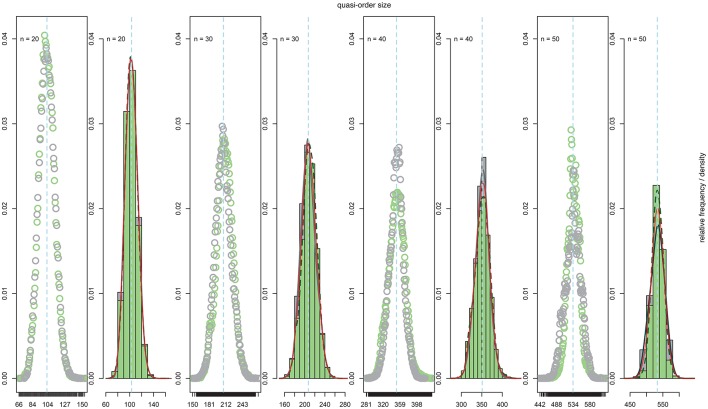
**For item numbers ***n*** = 20, 30, 40, and 50, pairs of scatter plots (left panel)** and histograms and kernel density estimates **(right panel)** of the sizes observed in the samples each of *N* = 500, 000 quasi-orders under the SIRM and STRM are presented (cf. Table 3).For all plots, duplicates were excluded. The *x*-axes stand for the observed quasi-order sizes (including the reflexive item pairs). Their relative frequencies or kernel and histogram density function values are placed on the *y*-axes. In the left panel scatter plots, the relative frequencies of the observed sizes for the SIRM method are shown in unfilled dark gray circles. For the STRM, unfilled light green circles are used. The right panel bell-shaped histograms for the SIRM and STRM are depicted in dark gray and light green colors, respectively. In the right panel kernel density representations, the Gaussian-like curves as the estimates of the size distributions under the SIRM and STRM are portrayed as solid dim gray and dashed dark green lines, respectively. The solid red lines plotted in the right panels are the graphs of the mean density functions averaged over both the SIRM and STRM kernel density estimates. The vertical dashed lines in light blue visualize the sample mean quasi-order sizes as the proxy and estimates of the true population means.

Exemplarily, the quasi-order samples of the size *N* = 500, 000 obtained for the item numbers *n* = 20, 30, 40, and 50 based on the SIRM and STRM procedures were further examined. In Figure 7, we present scatter plots, histograms, and kernel density estimates for the nuanced visualization. Figure 7 is arranged in pairs of plots. The plots of a pair refer to and are labeled with the same item number *n* ∈ {20, 30, 40, 50}. There is a left panel of scatter plots and a right panel containing kernel and histogram density estimates. The plots have the observed quasi-order sizes placed on the *x*-axes. Their relative frequencies or kernel and histogram density function values are placed on the *y*-axes. The results for the SIRM and STRM methods are seen in gray and green shades, respectively. The solid red lines in the right panels are the (pointwise) average density functions of the two kernel density estimates under the SIRM and STRM. The mean quasi-order sizes are added as vertical dashed lines in light blue. From Figure 7, we observe that for all of the item numbers considered, the distributions of the quasi-order sizes exhibit roughly Gaussian-like curves. Thus, it can be conjectured that this may also hold true in the corresponding populations of all possible quasi-orders.

## 7. Conclusion

### 7.1. Summary and final remarks

This paper has investigated how to randomly construct quasi-orders on finite sets such that a notion of representativeness for the process of sampling the discrete mathematical structures can be substantiated theoretically. An envisaged random process for quasi-order sampling must be feasible practically as well. It must be applicable in realistic settings when larger sets are used. For example, this is pertinent to the study of psychological or educational tests. Tests can be structured and efficiently employed based on quasi-orders. Quasi-orders on tests can be derived using data mining algorithms. Algorithms for mining quasi-orders have to be compared based on demanding simulation studies. In particular, Schrepp and Ünlü (2015) and Ünlü and Schrepp (2015) discussed the importance of representative random quasi-order samples needed in extensive simulation studies for the reliable comparison of data mining algorithms used to reconstruct relational dependencies among behavioral test items (cf. Section 1).

We have reviewed the state-of-the-art techniques currently available for quasi-order sampling (Section 2). For item numbers not greater than *n* = 15, the computations become prohibitively intensive. This can be attributed to the fact that the subsets of quasi-orders become quickly sparse with larger item numbers. However, in absolute terms, the quasi-order subsets are rapidly expanding in cardinalities. This situation is coupled with yet another problem. We have observed a substantial increase in variability of the constructed quasi-orders and of the summary statistics or estimates computed from the quasi-order samples for population parameters such as the mean size. Higher variability means greater imprecision. This may cause unstable estimation results. Two sources of variability seem to be effective in the present context. There is the typical *sampling variability*, that is, partial sample vs. complete population. From a combinatorial perspective, a second source of variation, termed *structural variability*, may entail effects on the computed aggregation measures. Structural variability is viewed as arising out of the deterministic order-theoretic constraints. Here, the transitivity constraint is imposed on the quasi-order as the sampled unit and an axiomatically defined mathematical object.

Thus, a general framework for a principled sampling theory for such mathematical structures as the quasi-orders will generally differ from the well-known statistical theory of survey sampling (e.g., Cochran, 1977; Thompson, 2012). In contrast to classical surveys (e.g., in the social or political sciences), sampling mathematical structures typically includes preparatory combinatorial work. For example, we have developed the discrete doubly inductive quasi-order construction. In particular, approaches similar to the simple random and stratified sampling techniques used in surveys have not been feasible or have been lacking in the context of sampling quasi-orders. In this paper, we have introduced variants of these basic survey techniques for the quasi-orders. Conceptually, the general idea comprises two building blocks that can also be applied to other discrete structures.

First, we have developed a combinatorial algorithm for incrementally constructing potentially all quasi-orders on a finite item set (Section 3). Proposition 2 shows that for any item number, the set of quasi-orders can be partitioned into specific constructive subsets.
Second, this deterministic procedure has been obtained probabilistically by randomization in the individual construction steps (Section 4). In the outer level inductive component, we have considered uniform random extensions of the trace quasi-orders to a higher dimension. We have combined this with an inner level inductive component to combinatorially correct the extensions that violate transitivity. The inner level deterministic corrections entail sampling biases. According to Proposition 4, the bias correction factors required for representative sampling can be derived.

Based on the correction factors, we have introduced three techniques for sampling quasi-orders (Section 5): the *absolute rejection method* (ARM), the *simple resampling method* (SIRM), and the *stratified resampling method* (STRM). These techniques have been compared with the *uniform extension method* (UEM) by Schrepp and Ünlü (2015). Analogous to the representativeness result for the UEM, Proposition 5 shows that for any item number, the bias-corrected hierarchical ARM procedure yields simple random sampling from the population of all quasi-orders. In extensive simulation studies (Section 6), we have demonstrated the usefulness of the sampling techniques for representative quasi-order generation. However, the ARM and UEM methods represent theoretical results. They become computationally intensive when larger item numbers are tried. We have seen that the conservative critical threshold for the ARM and UEM were *n* = 10 and *n* = 12 items, respectively. In contrast, the SIRM and STRM are the recommended procedures. They can be used with significantly higher item numbers. Within acceptable computing time, the SIRM and STRM methods have provided close to representative random quasi-orders on up to *n* = 50 items.

There are other characteristics than size that could be used to compare how representative the samples are. In Schrepp and Ünlü (2015), the quasi-order *width* (i.e., size of a longest anti-chain) and *height* (i.e., size of a longest chain) were used as the evaluation criteria to assess representativeness for the UEM method. Representativeness according to Definition 1 is assumed for arbitrary quasi-orders. Thus, we may infer that such a representative quasi-order sample will be unbiased for the population distributions of these and any other characteristic. In particular, based on the comparisons made of the SIRM and STRM with the UEM and ARM, we expect similar results for the evaluation criteria. As an example, for a set of *n* = 6 items, we compared representativeness for the UEM, ARM, SIRM, and STRM based on the quasi-order characteristics width, height, number of *maximal elements* (i.e., elements not in relation to any other element), and number of *minimal elements* (i.e., elements which no other element is in relation to). The average values are reported for 100 samples each of *N* = 1000 simulated quasi-orders.

The true *mean* values in the population of all quasi-orders on *n* = 6 items are 2.624 (width), 3.625 (height), and 1.899 (number of maximal elements = number of minimal elements). In respective order, for the UEM, the values (standard deviations in parentheses) are 2.626 (0.033), 3.623 (0.044), 1.898 (0.038), and 1.902 (0.046). For the ARM, we have 2.624 (0.032), 3.628 (0.040), 1.894 (0.041), and 1.900 (0.043), respectively. The respective values obtained under the SIRM are 2.623 (0.068), 3.619 (0.083), 1.899 (0.074), and 1.890 (0.087). The STRM yields 2.622 (0.069), 3.624 (0.076), 1.900 (0.076), and 1.896 (0.080), respectively.

### 7.2. Further research

What are interesting directions for future research? The SIRM and STRM sampling techniques were evaluated based on simulation. Theoretical work may study the probability theory foundation of these methods and of their interrelationship. With a finite sample size, the SIRM and STRM methods are approximate. Thus, further research may aim to investigate the large-sample or asymptotic properties of these resampling-based techniques. This could include quantifying the quality of approximation to representativeness and the development of related diagnostic error terms.

Variability reduction and the investigation of interval estimation techniques in the context of sampling quasi-orders are interesting directions for future research. Moreover, we have seen that the resulting graph for the mean quasi-order size, as a function of the item number, may be quadratic polynomial. We have also observed that the distributions of the quasi-order sizes are roughly bell-shaped. More in-depth analyses of these issues are needed.

Eventually, the discussion could be generalized to other combinatorial structures, which could include unlabeled (equivalence classes of) isomorphic quasi-orders and such special cases as weak, partial, or linear orderings.

## Author contributions

AÜ conceived the mathematical theory. AÜ and MS designed the software used in analysis. AÜ and MS wrote the paper. All the authors (AÜ and MS) reviewed the manuscript, approving the final version of the paper prior to submission.

### Conflict of interest statement

The authors declare that the research was conducted in the absence of any commercial or financial relationships that could be construed as a potential conflict of interest.

## Footnotes

^1^If we take the transitive closure of a rejected random reflexive relation, the result is a quasi-order. This *modified* entry-wise uniform sampling approach represents an *ad hoc* strategy. Although flexible, it lacks representativeness.The impact of forming the transitive closure of a random relation on the notion of representativeness, to our knowledge, seems to be an interesting and open problem for further research. Note that this problem cannot be answered with the current paper. The transitive closure used to correct for transitivity is *different* from the inductive correction procedure C introduced in Section 4.1. The transitive closure is the most parsimonious extension of a relation that additionally contains all indirectly accessible transitive pairs. The procedure C, however, can add and/or remove pairs according to the specific inductive routine to obtain transitivity. Thus, it is not obvious how a similar discussion can be elaborated for the transitive closure as a correction operator.

^2^For any *k* = 1, …, *n*, if the condition *C*_1_(*k*) holds true, we call the value 1 *admissible* for the entry *r*_*k, n*+1_. Similarly, for any *k* = 1, …, *n*, if *C*_2_(*k*) is satisfied, 0 is *admissible* for *r*_*k, n*+1_. In particular, *C*_1_(*k*) and *C*_2_(*k*) can be interpreted as follows. When filling an entry *r*_*k, n*+1_ at the *k*th position, only the previously filled entries at the positions 1, 2, …, *k* − 1 of the (*n*+1)th column are relevant. The possible values that may be chosen for the remaining unfilled entries of the added (*n*+1)th column and (*n*+1)th row need not be considered. For *k* = 1, the anchoring, *C*_1_(*k* = 1) and *C*_2_(1) are trivially satisfied. Thus, 0 and 1 are admissible for *r*_1, *n*+1_. In the construction process, *r*_1, *n*+1_ must take both values for the procedure to yield all of $r_{n}^{◇}$.

^3^Analogous to the top-down component, the three conditions can be interpreted as follows. For filling the entries *r*_*n*+1, *k*_ of the row *n*+1, only knowledge of the previously filled entries is relevant. Here, the previously filled entries are *r*_*n*+1, *i*_ for *i* = *k* + 1, …, *n* in the (*n*+1)th row [conditions *R*_1*b*_(*k*) and *R*_2_(*k*)] and *r*_*i, n*+1_ for *i* = 1, …, *n* in the (*n*+1)th column [condition *R*_1*a*_(*k*)].

^4^Note that with this procedure, we randomize the values filled into the positions *r*_1, *n*+1_, …, *r*_*n, n*+1_, *r*_*n*+1, *n*_, …, *r*_*n*+1, 1_, but not the order in which these positions are filled. A randomized order approach would probably make impossible the exhaustive discrete construction or would complicate the probabilistic extension. In the latter case, it may be difficult to correct for transitivity, and thus accompanied by induced sampling biases, even more intricate to determine the required bias correction factors. In Proposition 4, we will see that this is possible, and relatively straightforward, for the fixed order approach of this paper.
